# Age- and sex-divergent neurodevelopmental patterns revealed through structural magnetic resonance imaging features

**DOI:** 10.3389/fnana.2026.1906618

**Published:** 2026-09-21

**Authors:** O. Kabenova, R. Zhulduzbayev, I. Brak, B. Kerimkulov, R. Siugzdaite, A. Kustubayeva

**Affiliations:** 1 Department of Biophysics, Biomedicine and Neuroscience, Al-Farabi Kazakh National University, Almaty, Kazakhstan; 2 Kazakh-British Technical University, Almaty, Kazakhstan; 3 National Centre for Neurosurgery, Nur-Sultan, Kazakhstan; 4 Department of Artificial Intelligence, RUDN, “Peoples’ Friendship University of Russia named after Patrice Lumumba”, Moscow, Russia; 5 Sunkar Medical Center, Almaty, Kazakhstan; 6 Ghent University, Ghent, Belgium; 7 KULeuven, Leuven, Belgium; 8 Brain Institute, Almaty, Kazakhstan

**Keywords:** age prediction, brain maturation, machine learning, neurodevelopment, structural MRI

## Abstract

**Introduction:**

Brain maturation during childhood and adolescence involves complex structural changes that vary among individuals. Characterizing these trajectories is essential for understanding normative development and identifying deviations associated with neurological conditions.

**Methods:**

We analyzed structural magnetic resonance imaging data from 179 healthy participants aged 7–20 years. Cortical thickness, regional surface area, and regional brain volumes were assessed to characterize age-related morphometric patterns and sex differences. These features were also used to predict chronological age. Ten regression algorithms spanning regularized linear, Bayesian, kernel-based, and gradient-boosting approaches were evaluated using repeated age-stratified five-fold cross-validation.

**Results:**

Total gray matter volume decreased with age, whereas white matter volume increased, reflecting ongoing myelination and brain maturation. The caudate nuclei and nucleus accumbens showed age-related volume reductions, while cerebellar and brainstem volumes increased, suggesting continued maturation of motor and sensory systems. No significant age-related associations were observed for cortical surface area. In contrast, cortical thickness showed widespread age-related decreases, including in the superior temporal sulcus, anterior cingulate cortex, and prefrontal cortex, consistent with functional specialization. Males had significantly larger cortical, subcortical, and white matter volumes, as well as larger surface areas across multiple cortical regions. However, cortical thickness did not differ significantly by sex. A stacking ensemble achieved the best age-prediction performance, with an age-bias-corrected mean absolute error of 1.41 years. Despite the observed morphological sex differences, the corrected brain-age gap did not differ significantly between sexes.

**Discussion:**

These findings provide a region-specific reference for normative brain development in a Central Asian pediatric and adolescent sample and establish a preliminary framework for predicting age from standard brain morphometric measures. Independent replication in larger and more diverse samples is required to assess the generalizability of these findings.

## 1 Introduction

Most large-scale neurodevelopmental MRI studies have been conducted in Western populations, whereas structural neuroimaging data from Central Asian pediatric and adolescent cohorts remain limited. The present study aims to address this gap by characterizing age- and sex-related morphometric patterns in a cohort from Kazakhstan and evaluating whether structural MRI features can support chronological age prediction.

Significant progress has been made over the last 36 years in understanding the brain’s regional morphology and function during adolescence. Major morphological and functional changes occur in the human brain during maturation (Giedd et al., 1999). Magnetic resonance imaging (MRI) has emerged as a powerful tool for investigating brain development, enabling the non-invasive characterization of structural and functional changes across development. Structural MRI (sMRI), diffusion tensor imaging (DTI), and functional MRI (fMRI) allow researchers to study changes in both gray (GM) and white matter (WM) throughout development. MRI studies have provided new insights into how different brain regions develop at varying rates, highlighting the importance of brain network maturation and regional specialization during key developmental windows (Giedd et al., 1999). The human brain undergoes dynamic structural changes throughout development, which continue into early adulthood and beyond. Maturation is a complex, dynamic process that spans from early childhood into adulthood, involving significant structural and functional changes that influences development of sensory, cognitive, and motor abilities. Maturation is characterized by a wide range of processes, including myelination, synaptic pruning and therefore changes in cortical thickness. Understanding the trajectory of these developmental processes is essential for establishing typical brain growth patterns and identifying deviations that may underlie neurodevelopmental disorders.

By the age of six, the volume of the brain reaches approximately 90% of the volume of the adult brain (Reiss et al., 1996). The thickness and volume of the cerebral cortex has a nonlinear character with a U-shaped course of maturation, with an increase by 7–10 years and later with gradual decrease, subsequently coming to stabilization (Casey et al., 2000; Gogtay et al., 2004; Shaw et al., 2008). Intensity of maturation in brain areas differs from each other: more simple three-layered parts of the cortex undergo simple linear changes, while more complex six-layered of the cortex have rather complex development trajectories (Tamnes et al., 2010). Maturation of the cortex goes in the direction from posterior regions to anterior parts, as well as from the periphery to the center (Shaw et al., 2008). According to Brown et al. (2012) cortical areas are increasing until 12.3 years old, the volume of the hippocampus – until 14.2 years old and following decrease in the volume; the volume of thalamus increases until 17.8 years old and comes to a plateau at 20 years old (Brown et al., 2012). In studying MRI of normal brain development from ages 4 to 18, researchers note that total brain volume is significantly larger in males compared to females (Brain Development Cooperative Group, 1991). Harvey and colleagues found that the relationship between brain size and body size in humans is surprisingly weak (Harvey and Krebs, 1990). However, it was established that total brain volume (TBV) is 12% higher in males than in females. At the same time, early reduction in cortical gray matter (CGM) volume in the sensorimotor zone and later in the dorsolateral prefrontal cortex does not depend on sex differences (Giedd, 2004). It was also found that brain development follows the same pattern in both females and males, although, on average, females develop slightly earlier (Brouwer, 2021). Lange et al. (1997) studying MRI of normal brain development from ages 4 to 20, identified a small systematic association of brain tissue volumes with body mass index (BMI), but not with IQ, family income, or parental education. It was also established that head circumference is an insufficient indicator of brain volume in typically developing children and adolescents. Mills et al. found evidence of continued changes in both intracranial volume and total brain volume during adolescence, though following different trajectories. Their results indicate that cortical GM volume peaks in childhood and gradually declines through the second decade of life, slowing during the third decade, while white matter increases intensely until mid-to-late adolescence before growth rate slows. They also noted that adjusting for brain size influences regional brain development patterns, particularly with regard to sex differences (Mills et al., 2016).

Based on the results of the longitudinal study during brain development from ages 4 to 24, Giedd et al. (1999) showed a linear increase in WM volume, whereas changes in cortical gray matter (GM) volume were nonlinear, demonstrating a tendency to increase until adolescence and then decrease during the post-adolescent period. Interestingly, different lobes of the GM reached their peak volumes at different ages: in the frontal and parietal lobes, the relative maximum was reached by age 12, in the temporal lobe by about age 16, and in the occipital lobe growth continued up to age 20. Lange et al. identified age-related features of cortical GM volume reduction and WM increase. At the same time, left-sided asymmetry was observed in the occipital cortex GM and WM, temporal WM, caudate nucleus, and globus pallidus, while right-sided asymmetry was identified in temporal cortex GM (Lange et al., 2010). Conducting a large-scale study using fully automated analytical approaches and MRI data from 200 healthy children, researchers concluded that global developmental patterns were evident in both gray and white matter, with gray matter decreasing and white matter significantly increasing with age. Gray matter loss was mostly pronounced in the parietal lobe and least pronounced in the cingulate gyrus and posterior temporal regions. White matter volume increase with age was almost uniform, with emphasis on the pyramidal tract. Sex differences were observed for both gray and white matter. The analysis confirmed significant sex differences in brain morphology, such as larger amygdala volume in boys and larger caudate volume in girls (Wilke et al., 2008).

Within the developmental trajectory, sex has emerged as a significant biological variable influencing the timing, pattern and pace of brain maturation. Females have been found to be more advanced in their development of brain structure (Gennatas et al., 2017; Giedd et al., 1999; Koolschijn and Crone, 2013). Numerous neuroimaging studies have demonstrated that males and females follow distinct developmental trajectories in several brain regions. For example, males typically exhibit larger total brain volumes than females throughout development, however females have a tendency to reach peak in cortical gray matter volume earlier (Lenroot et al., 2007). Furthermore, differences have been observed in maturation of prefrontal cortex, limbic structures and corpus callosum, which are involved in executive functions, social cognition and emotional regulation (Blakemore et al., 2010; Peper et al., 2011). These anatomical differences may underlie observed sex differences in behavior, cognitive abilities, and susceptibility to psychiatric disorders. Investigating brain development trajectories, Wierenga et al. established that in most regions, cortical thickness decreased linearly with age, while cortical volume and surface area showed curvilinear trajectories. On average, maximum in cortical surface area was reached later than maximum in cortical volume during development. Sex differences were more pronounced in cortical volume and surface area than in average thickness (Wierenga et al., 2014). Ducharme et al., studying developmental trajectories, found that most brain regions showed linear reduction in cortical GM thickness, with several areas - mainly in the temporoparietal regions of both hemispheres and in the right prefrontal cortex - reaching peak thickness at age 8 or earlier. The only gender difference was in faster thinning of the occipital areas in boys compared to girls. According to their data, the average annual reduction in mean cortical GM thickness was 0.027 mm (Ducharme et al., 2015). Investigation of the effects of hemisphere, sex, and age on brain size variability in brain regions among 115 healthy children and adolescents aged 4–20 years revealed that structural variability differed across regions, with lateral ventricles showing the highest coefficient of variation and putamen the lowest. In boys, differences were significantly higher in the left hemisphere and left superior temporal gyrus compared to girls, whereas in the caudate nucleus and putamen of the right hemisphere, girls showed greater variability than boys (Lange et al., 1997). Age-related effects manifested as increased variability after puberty in lateral ventricles, hippocampus, and superior temporal gyrus.

In this study, we expect to find significant association between age and structural brain development, specifically showing widespread cortical thinning during childhood and adolescence across multiple brain regions. Second, we investigate the independent contributions of regional brain volumes, cortical thickness, and surface area to age-related brain changes, aiming to understand which features most strongly reflect developmental trajectories. Finally, we investigate whether sex differences influence structural brain maturation, expecting that developmental patterns may differ between males and females in terms of timing and regional specificity. Further we examine whether multivariate combinations of these morphometric features can predict chronological age and whether the resulting brain–age gap differs by sex.

Beyond mass-univariate associations, the multivariate pattern of MRI features can be used to estimate an individual’s chronological age, a task referred to as brain-age prediction. The discrepancy between an individual’s chronological and predicted age, the brain-age gap (BAG), has emerged as a sensitive biomarker of typical and atypical neurodevelopment (Cole and Franke, 2017; Franke et al., 2010). Recent systematic comparisons indicate that no single estimator is universally optimal: regularised linear models, Bayesian regression, kernel methods and gradient-boosted decision trees all attain competitive performance on features, and multi-modal stacking ensembles often improve upon any single estimator (More et al., 2023; Peng et al., 2021). In the present study we therefore evaluate a panel of complementary algorithms in an age-stratified, repeated cross-validation framework, with explicit assessment of sex-related effects. We additionally apply the bias-correction scheme of Beheshti et al. (2019) and Smith et al. (2019) to remove the regression-to-the-mean artefact that systematically distorts raw brain-age estimates (Beheshti et al., 2019; Smith et al., 2019). By integrating these modern methodological standards, we aim to characterise both the accuracy with which adolescent age from cortical and subcortical morphometry and the anatomical features that most strongly carry that information.

## 2 Materials and methods

### 2.1 Participants

A total of 223 participants of population from Kazkahstan were initially recruited for the study. Children’s caregivers were contacted by phone and invited to participate in the study to complete a structural MRI scanning at Sunkar Medical Center (Almaty, Kazakhstan). Participants were required to meet eligibility criteria to participate in the study. All participants had normal or corrected-to-normal vision and had no history of psychiatric or neurological disorders. Additional exclusion criteria included substance dependence, history of brain injury, previous loss of consciousness for greater than ten minutes and any contraindications for MRI. All participants provided written informed consent prior to their inclusion in the study. For the participants aged under 18 the consent was provided by their caregivers. The study protocol was approved by the Ethics Committee of al-Farabi Kazakh National University.

44 participants were excluded due to voluntary withdrawal or wearing braces during the MRI scanning procedure. Participants whose MRI scans contained motion or other imaging artifacts were rescanned to obtain images of sufficient quality for analysis. Post-scanning quality check was also performed in three steps: (1) Outlier Detection, (2) Internal Surface Method, (3) External Surface Method. The final sample consisted of 179 healthy participants (aged 7–20 years; mean age: 13.9 years; SD: 4.03 years), including 88 females (49%). Handedness was assessed by Annett (1970). Body Mass Index (BMI) was calculated as weight in kilograms divided by height in meters squared (kg/m^2^). Height and weight were measured using a calibrated stadiometer and digital scale, respectively, with participants wearing light clothing and no shoes. Controlling for BMI helps isolate age- and sex-related effects on brain structure from those potentially influenced by variations in body mass (Table 1).

**Table 1:** Demographic characteristics of the study sample.

Demographic characteristic	Sex	
	Male	Female
Sample size (*N* = 179)	91	88
Age (years)^a^	13.91 (4.00)	13.82 (4.08)
Intracranial volume (mm3)	1,447,126 (125000)	1,602,759 (126000)
Right handed (%)	85.43 (0.03)	90.17 (0.02)

^a^Mean (SD).

### 2.2 MRI data acquisition

MR Imaging for this cohort was performed using a 48-channel phased-array head coil on a 3 T GE Signa Architect MR Scanner (GE Healthcare). T1-weighted images were acquired in the sagittal plane using a 3D BRAVO sequence (frequency FOV = 240 mm, repetition time (TR) = 7.716 ms, echo time (TE) = 3.14 ms, slice thickness = 1.2 mm, slice gap = 0.6136 mm, and voxel size = 0.4688 × 0.4688 × 1.2 mm). A total of 260 sagittal slices were collected (matrix 137 size = 512 × 512).

### 2.3 MRI data analysis

#### 2.3.1 MRI data preprocessing

All MRI data were processed individually through the automated subcortical segmentation and cortical parcellation tool for a surface-based approach in FreeSurfer, version 7.2.0 (Harvey and Krebs, 1990)^1^. Quality check was done in three steps: (1) Outlier Detection, (2) Internal Surface Method, (3) External Surface Method. To increase the probability that each discrete region is correctly labelled, FreeSurfer considers aspects of the collected data and general aspects of MRI such as signal intensity of different regions, producing accurate and reliably segmentation. We extracted 68 indices of surface area and 68 indices of cortical thickness using DK atlas, and 15 indices of regional volume for subcortical structures, resulting in total of 151 unique neuroanatomical features. White matter, gray matter, and pial matter boundaries were reviewed in each subject for gross artifacts and errors in segmentation by the authors. These 151 measures were used for the mass-univariate analyses of age and sex effects in Sections 2.3.2 and 3.1–3.2. Brain-age prediction used a separate, extended set of features assembled from the same FreeSurfer outputs in Section 2.4.1.

#### 2.3.2 Statistical data analysis

Statistical analysis was carried out using RStudio (Version 2024.04.2). Pearson’s correlation was used to investigate association between neuroanatomical features of structural MRI and age. We preselected 68 cortical regions and 16 subcortical regions to investigate correlations with age and corrected for multiple comparisons using Bonferroni correction to control for the increased chance of Type I errors (false positives). Gender differences in regional brain measures were assessed using linear regression models, with each regional measure as the dependent variable and gender as the main predictor. Only cortical volume was used as a proportion of total ICV in the analysis. Age and intracranial volume (ICV) were included in a model as covariates to account for potential differences related to age and overall head size. Brain differences were visualized using ggseg() package in RStudio (Vidal-Piñeiro et al., 2019).

### 2.4 Machine learning framework

The goal of the machine-learning analysis was to predict chronological age from morphometric MRI features and to characterise both the precision of that prediction and the regional features that drive it. All analyses were performed in Python 3.11 using scikit-learn 1.2 (Pedregosa et al., 2011), CatBoost 1.2 (Prokhorenkova et al., 2018), LightGBM 4.6 (Ke et al., 2017), XGBoost 3.0 (Chen and Guestrin, 2016), Optuna 4.8 (Akiba et al., 2019) and SHAP 0.45 (Lundberg and Lee, 2017).

#### 2.4.1 Morphometric feature matrices

For brain-age prediction, feature matrices were assembled from the FreeSurfer outputs described in Section 2.3.1. Univariate analyses in Sections 2.3.2 and 3.1–3.2 used 151 measures, including 68 regional surface-area indices, 68 regional thickness indices, and 15 predefined subcortical volume indices. The prediction matrices below extend this univariate set.

The Area matrix comprised the 68 regional surface-area measures together with estimated intracranial volume and four hemisphere-level summary measures, yielding 73 features in total. The Thickness matrix comprised the 68 regional thickness measures plus the same five global covariates, also yielding 73 features. Regional cortical thickness and surface area were retained in raw form, with ICV and hemisphere summaries included as separate predictors so that overall brain scale could be modelled explicitly.

The Volume matrix comprised the same 15 subcortical volume indices used in Section 2.3.1, supplemented with additional global and regional compartment volumes exported from FreeSurfer, including tissue summaries, ventricles, cerebellum, and corpus callosum segments. Subcortical and other volumetric features in this matrix were expressed as proportions of intracranial volume by dividing each volume by the participant-specific ICV estimate. This reduced dependence on absolute head size while retaining regional volumetric information. Columns with more than 43% missing data were removed, leaving 50 volume features.

Sporadic missing values, 25 cells in the Area matrix and one cell in the Volume matrix, were imputed with the column-wise median. A fourth matrix, Concat, was formed by horizontally concatenating the three modality-specific matrices, yielding 196 features per participant. All matrices were standardised to zero mean and unit variance inside each cross-validation fold for the linear, Bayesian, kernel and Gaussian Process models. The gradient-boosting models received the raw values.

We used this preprocessing scheme because age prediction requires joint use of regional morphology and overall brain scale. Univariate sex comparisons in Sections 3.1 and 3.2 followed the ICV adjustment strategy described in Section 2.3.2.

#### 2.4.2 Machine learning models

Ten regression algorithms from four methodological families were compared. We had up to 196 morphometric features but only 179 participants, so every model had to limit overfitting while still allowing for non-linear and multi-modal age effects during adolescence.

Lasso (Tibshirani, 1996), ElasticNet (Zou and Hastie, 2005), and Ridge (Hoerl and Kennard, 1970) were our first choice for this setting. Adolescent morphometric data contain many correlated measures relative to sample size, especially among left–right homologous regions, and in the Concat matrix features outnumber participants. These models limit overfitting while reflecting different views of how age is encoded in the brain. Lasso selects a sparse set of age-informative regions. ElasticNet remains stable when many features covary. Ridge retains all features and shrinks their influence, allowing distributed age-related effects across the brain. All three are interpretable, which matters when linking predictions back to anatomy.

BayesianRidge (MacKay, 1992) fits the same high-dimensional problem but learns how strongly to shrink coefficients from the data and returns an uncertainty interval around each prediction. That is useful in a developmental sample where single morphometric measures are imperfect and correlated.

We also asked whether adolescent maturation might follow smooth but non-linear trajectories that linear models miss. KernelRidge and a Gaussian Process Regressor (GPR) with an RBF kernel address this while remaining regularised. KernelRidge is a lighter non-linear extension of Ridge. GPR is a fully Bayesian non-linear baseline and a standard reference in brain-age work (Franke et al., 2010; Rasmussen and Williams, 2006).

LightGBM (Ke et al., 2017), XGBoost (Chen and Guestrin, 2016), and CatBoost (Prokhorenkova et al., 2018) can capture interactions among features without hand-built terms, which may matter when area, thickness, and volume contribute jointly. Tree models can overfit small cohorts, so we tuned them with nested cross-validation in Section 2.4.4.

Finally, we combined Area, Thickness and Volume in a stacking ensemble because each modality reflects a different aspect of maturation, like surface scale, cortical thinning, and subcortical/global volume change. For each modality, we took out-of-fold predictions from Lasso, the best linear model on that matrix, and fed them to a Ridge meta-learner. Because the meta-learner only sees OOF inputs, no test-fold information leaks into training, and the reported performance remains valid (More et al., 2023).

#### 2.4.3 Cross-validation protocol

Out-of-fold predictions were obtained with RepeatedStratifiedKFold (five folds, two repeats; 10 training/validation splits in total). The stratification variable was a five-bin discretisation of chronological age obtained with pandas.qcut, which guarantees that each fold contains a representative spread of the 7–20 year range. In sex-specific analyses the same scheme was applied separately within each sex, with four age bins to keep each stratum populated. All preprocessing was encapsulated within a scikit-learn Pipeline and refitted inside every training fold, so that no test-set information leaked into the training data.

#### 2.4.4 Hyperparameter optimisation

Hyperparameters of the gradient-boosting models were tuned with the Tree-structured Parzen Estimator (TPE) implemented in Optuna (Akiba et al., 2019). For each model, 20 trials were drawn and evaluated through an inner three-fold cross-validation that was nested within the outer 5×2 cross-validation (CV). The TPE sampler concentrates trials in regions of the search space that have produced low validation errors in previous trials, which is more sample-efficient than random or grid search. The search spaces covered the number of estimators (100–600), learning rate, tree depth or number of leaves, L1/L2 regularisation, sub-sampling and feature sub-sampling.

#### 2.4.5 Performance metrics and statistical comparison

Four complementary metrics were computed on the concatenated OOF predictions of each model. The mean absolute error (MAE in years), the coefficient of determination R^2^, the Pearson correlation coefficient r between predicted and chronological age, and the median absolute error (MedAE). MAE and MedAE were preferred for clinical interpretation as both are expressed in years and are robust to outliers. R^2^ and r quantify rank ordering and explained variance.

We additionally report the proportion of participants for whom the absolute prediction error did not exceed one year and two years. These figures tell us in what fraction of individuals does the model estimate age to within k years and are the regression analogue of the accuracy and sensitivity reported for classification studies. Conventional thresholds in the brain-age literature are MAE < 5 years for adult cohorts spanning several decades (Cole and Franke, 2017; Bashyam et al., 2020) and MAE < 2 years for narrower adolescent samples (Khundrakpam et al., 2015; Ball et al., 2021).

Ninety-five-percent confidence intervals (CI) for MAE and R^2^ were estimated by 1,000 non-parametric bootstrap resamples of the OOF predictions. Pairwise comparisons between models were performed with the two-sided Wilcoxon signed-rank test on the per-participant absolute errors. Sex-related differences in the bias-corrected BAG were tested with the two-sided Mann–Whitney U test, which makes no distributional assumption.

#### 2.4.6 Age-bias correction

Regression-based brain-age estimators systematically overestimate age in younger and underestimate age in older participants, a regression-to-the-mean artefact unrelated to neurobiology (Le et al., 2018; Smith et al., 2019). To remove this artefact we applied the procedure of Beheshti et al. (2019). The OOF predictions were regressed against chronological age to obtain a slope a and intercept b. For each participant, the corrected prediction was defined as pred_corrected = pred − (a × age + b − age), where pred is the out-of-fold predicted age and age is that participant’s chronological age.

We obtained the slope and intercept by regressing all pooled out-of-fold predictions on chronological age for the full sample. We then applied this single correction line to every participant. Corrected performance metrics in Table 2 and the bias-correction figures use the same pooled out-of-fold set. This is common post-hoc calibration. It can be slightly optimistic compared with a nested correction that fits bias parameters only on training folds and applies them to held-out folds. Raw out-of-fold metrics are not affected by this step.

**Table 2:** Stacking-ensemble performance before and after age-bias correction.

Group	Prediction	n	MAE, y	R^2^	Pearson r	±1 y, %	±2 y, %
Overall	Raw	179	1.55	0.76	0.87	42.5	65.4
Overall	Corrected	179	1.41	0.81	0.92	40.8	71.5
Female	Raw	88	1.53	0.77	0.88	43.2	67.0
Female	Corrected	88	1.39	0.82	0.92	42.0	73.9
Male	Raw	91	1.57	0.75	0.87	41.8	63.7
Male	Corrected	91	1.43	0.81	0.92	39.6	69.2

All metrics are reported on both the raw and the bias-corrected predictions. The corrected BAG is the quantity that should be carried forward into any subsequent group comparison.

Bias-corrected out-of-fold predictions were used for all downstream analyses. These included computation of the brain-age gap and all group comparisons reported in Section 3.3.3. Raw predictions were retained only for before-and-after performance summaries in Table 2 and Figure 1.

**Figure 1:**
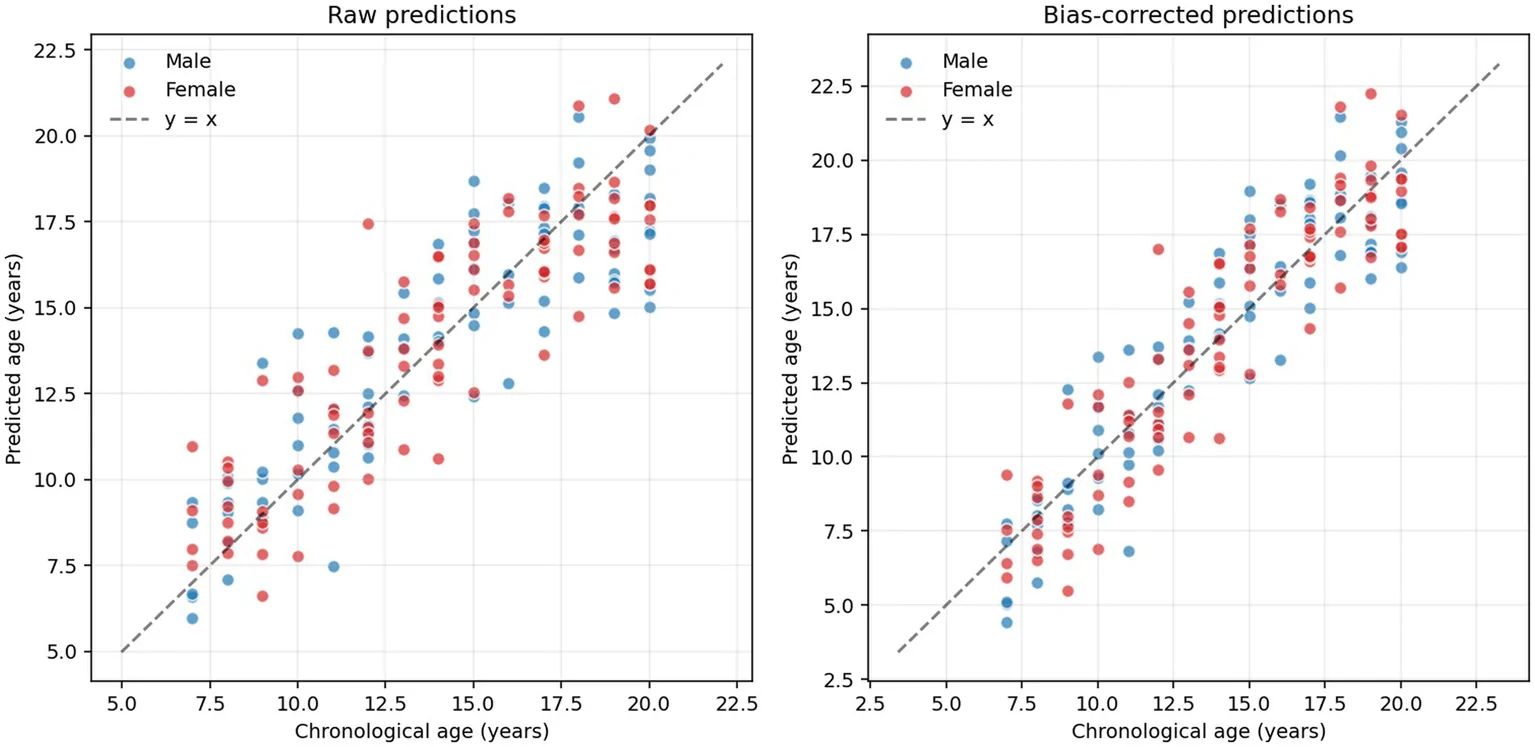
Predicted versus chronological age. The left panel displays raw model predictions. The right panel displays predictions after Beheshti bias correction. On both panels, the horizontal axis is chronological age in years. The vertical axis is predicted brain age in years. Each point represents one participant. The dashed gray line is the identity line where predicted age equals chronological age. Points above this line indicate overestimation. Points below this line indicate underestimation. Females are shown in red and males in blue.

## 3 Results

### 3.1 Age differences

#### 3.1.1 Correlation analysis between volume of regional brain areas and age

Pearson correlation analysis was performed to explore the relationships between age and the volumes of different brain regions. The results revealed several significant correlations, particularly in regions associated with aging and structural brain changes.

A significant negative correlation was found between age and left, right hemisphere and total cortical volume (r = −0.504, *p* < 0.01; r = −0.493, *p* < 0.01; r = −0.499, *p* < 0.01), indicating a decline in cortical volume with increasing age. Total gray matter volume also displayed a significant negative correlation with age (r = −0.434, *p* < 0.001). These findings indicate lower cortical volume measures in older participants; cortical thickness associations were evaluated separately. Age was positively correlated with left (r = 0.309, *p* < 0.01), right cerebral white matter (r = 0.316, *p* < 0.01), and total cerebral white matter volume (r = 0.313, *p* < 0.01) showing significant positive correlations. (Figure 2.) Central corpus callosum was also significantly correlating with age (r = 0.299, *p* < 0.001). Moreover, age was positively correlated with left and right cerebellum WM (r = 0.309, *p* < 0.001; r = 0.311, *p* < 0.001) indicating age-related increase in cerebellar volume which is consistent with previous longitudinal studies describing continued cerebellar maturation during childhood and adolescence (Tamnes et al., 2017).

**Figure 2:**
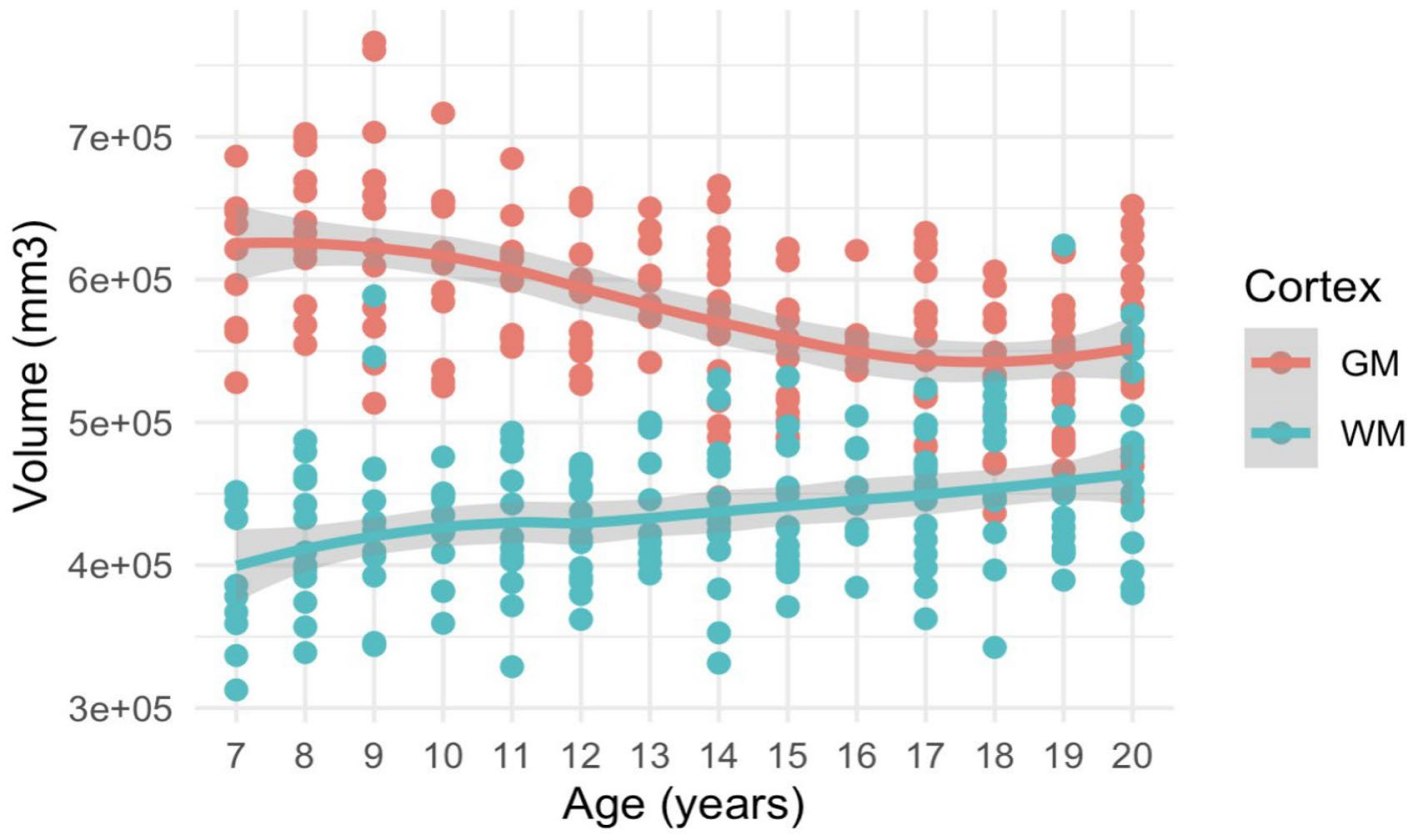
Age-related trajectories of cortical GM and WM volumes from 7 to 20 years. The *x*-axis represents age in years, and the *y*-axis represents cortical volume in mm^3^. Points indicate individual measurements, while fitted curves illustrate age-related trends in GM and WM volumes.

Left and right ventral DC was positively correlated with age (r = 0.368, *p* < 0.001, r = 0.311, *p* < 0.001). The significant negative correlations observed between age and the right caudate nuclei (r = −0.247, *p* < 0.01), the left (r = −0.397, *p* < 0.01) and right (r = −0.424, *p* < 0.01) accumbens areas. The age-related reduction in nucleus accumbens volume may be consistent with previously described maturational processes, including synaptic remodelling and tissue reorganization (Mills et al., 2016). However, because the present study is cross-sectional, the underlying biological mechanisms cannot be determined directly. The positive correlation between brainstem volume and age (r = 0.445, *p* < 0.01) and third ventricle and age (r = 0.332, *p* < 0.001) were observed, that is also in line with normal growth, myelination, motor/sensory development. The complete correlation results are provided in the Supplementary Table S1.

#### 3.1.2 Correlation analysis of cortical thickness and age

We conducted Pearson correlation analysis to examine the relationship between cortical thickness across various brain regions and age. Significant correlations (*p* < 0.05), adjusted for multiple comparison were observed in several regions, indicating both positive and negative relationships between cortical thickness and age (Figure 3).

**Figure 3:**
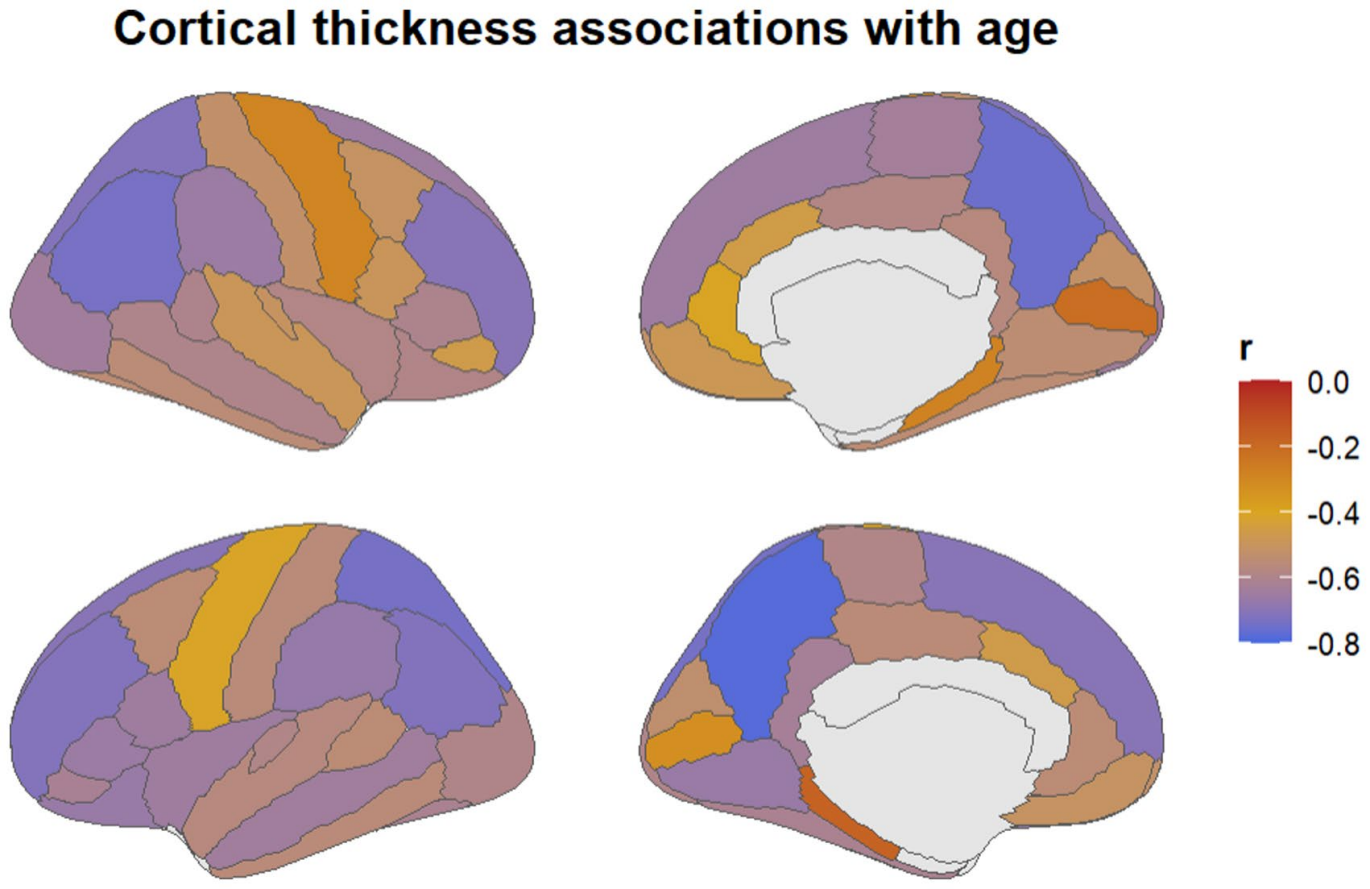
Associations between cortical thickness and age across cortical regions. The maps show regional correlation coefficients (*r*) *be*tween cortical thickness and age across the studied age range. Colors represent the strength and direction of the correlations according to the color scale, with values ranging from 0 to −0.8. More negative *r val*ues indicate stronger age-related decreases in cortical thickness. Lateral and medial views of both cerebral hemispheres are presented.

The majority of brain regions showed significant negative correlations with age, that suggests age-related cortical thinning during childhood and adolescence. Both left (r = −0.569, *p* < 0.001) and right (r = −0.487, *p* < 0.001) superior temporal sulcus (STS) exhibited a strong negative correlation. Significant negative cross-sectional associations between age and cortical thickness were observed in the left and right caudal anterior cingulate cortex (ACC) (r = −0.461, *p* < 0.001) and (r = −0.455, *p* < 0.001) respectively was observed as well. The left (r = −0.549, *p* < 0.001) and right (r = −0.508, *p* < 0.001) caudal middle frontal cortex, part of the prefrontal cortex, showed a significant decrease in thickness with age. The visual processing region, the left (r = −0.533, *p* < 0.001) and the right (r = −0.516, *p* < 0.001) cuneus, displayed cortical thinning with age. Both right (r = −0.700, *p* < 0.001) and left (r = −0.712, *p* < 0.001) rostral middle frontal cortex showed significant negative correlations. Cortical thinning in these regions may reflect increasing specialization and efficiency in language, executive function and self-regulation. Only 6 regions did not show significant correlations with age (left and right entorhinal cortex, left parahippocampus, right pericalcarine area, right and left temporal pole). Full information on corelation analysis is in Supplementary Table S3.

#### 3.1.3 Correlation analysis of cortical area and age

There were no significant associations found between cortical surface area of the different brain regions and age after correction for multiple comparisons (Supplementary Table S2).

#### 3.1.4 Developmental trajectories of global cortical phenotypes

Total cerebrum volume and cortical surface area appeared largely stable from age 7 onward, with males consistently showing higher values than females in this sample, suggesting that global cortical scale may already be largely established by early adolescence. Mean cortical thickness showed a different developmental pattern. Both sexes demonstrated progressive cortical thinning, although females started from slightly higher global mean values and males declined more steeply, resulting in convergence of the trajectories around 13–14 years. These sex-stratified thickness curves describe this cohort only. They should not be interpreted as general normative developmental trajectories. They reflect cohort-level mean curves rather than systematic regional sex differences. Region-by-region comparisons in section 3.2 revealed no significant sex effects in cortical thickness after multiple-comparison correction. A transient acceleration of thinning in males around 12–13 years was also observed and may plausibly reflect pubertal-related change in this sample. This interpretation remains tentative given the cross-sectional design and sample size. However, given the small per-age sample size (n ≈ 5) and the derivative-based smoothing procedure, this feature should be interpreted cautiously rather than as definitive evidence of pubertal acceleration. Between-subject variance for volume and surface area remained relatively stable across age, whereas cortical thickness variability showed greater age dependence. At the youngest end of the age range, however, the variance and rate-of-change panels should be interpreted cautiously. Bootstrap confidence intervals widened substantially between 7 and 11 years, and for surface area in females the variance estimate transiently dropped below zero at age 7, most likely reflecting sparse sampling rather than a biological effect (Figure 4).

**Figure 4:**
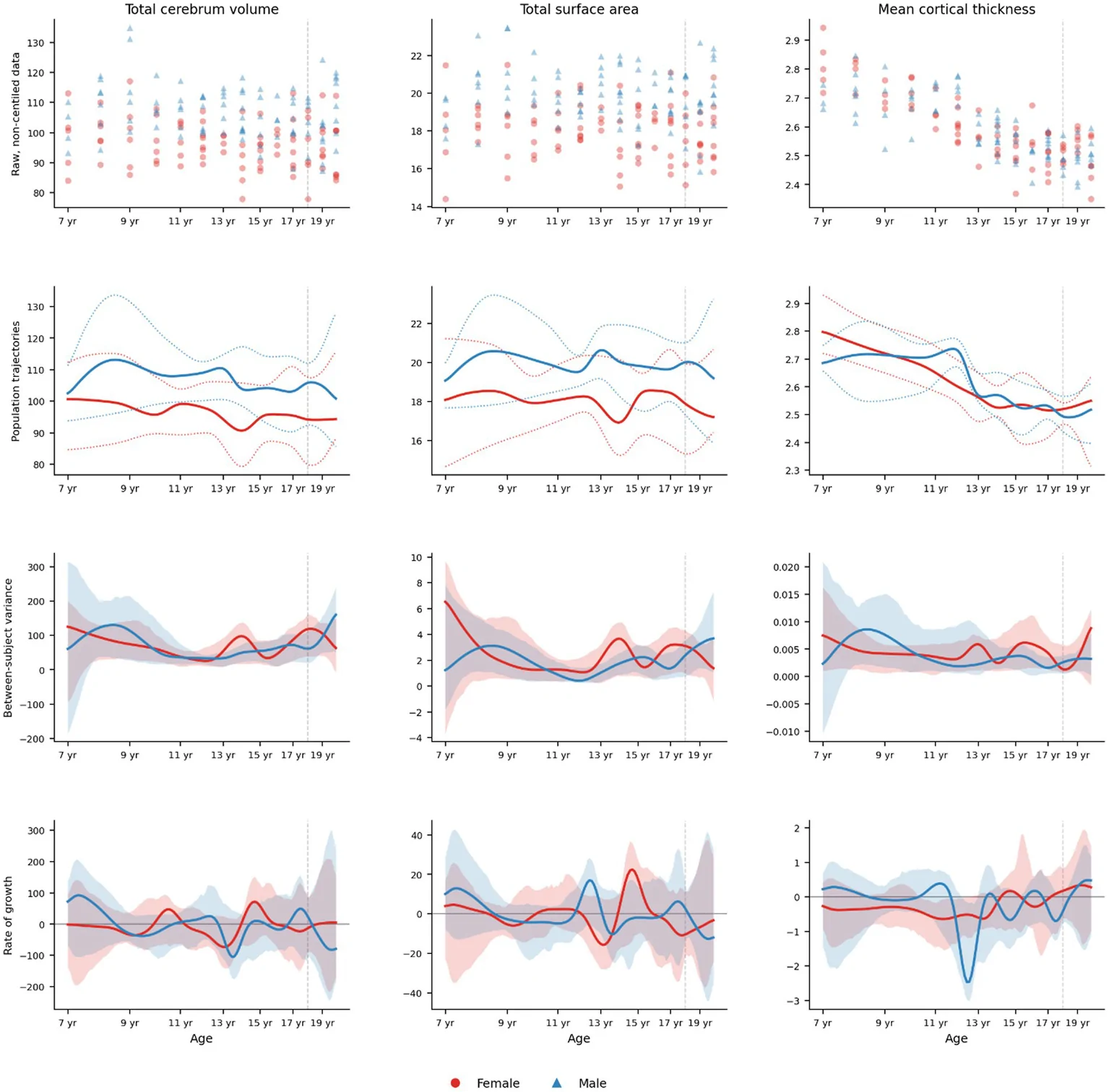
Sex-specific developmental trajectories of global cortical morphometric measures from 7 to 20 years. The columns show total cerebrum volume, total cortical surface area, and mean cortical thickness, respectively. The first row presents individual raw non-centered measurements by age. The second row shows estimated population trajectories with dotted lines indicating uncertainty intervals. The third row depicts age-related between-subject variance, with shaded areas representing uncertainty intervals. The fourth row shows the estimated rate of growth (first derivative) across age, with the horizontal zero line indicating no change. Females are shown in red and males in blue.

Rates of change were estimated as the first derivatives of sex-stratified, smoothed median trajectories. In normative brain-chart studies, developmental change is often summarized in this way from median age curves. However, here we used a descriptive binning–smoothing pipeline rather than Generalized Additive Models for Location, Scale, and Shape (GAMLSS), given N = 179, ages 7–20 years, and a cross-sectional sample. For each sex and global metric, age was transformed as log(1 + age). Medians were computed in quantile-based age bins (≥3 subjects per bin, 10 bins for the primary fit), interpolated with Piecewise Cubic Hermite Interpolating Polynomial (PCHIP) method to smoothly estimate values between observed data points, lightly smoothed with a Gaussian filter (σ = 2). Rates were then obtained as numerical derivatives with respect to log(1 + age) and smoothed again (σ = 2), so they reflect change on the log-age scale rather than change per calendar year. Shaded bands show 2.5–97.5% bootstrap intervals (400 resamples, 8 bins per resample). These trajectories describe our cohort only and should not be interpreted as normative lifespan charts (Figure 4). Variance and rate estimates were least stable between 7 and 11 years, when fewer participants were available at each age.

### 3.2 Sex differences

#### 3.2.1 Volume

Significant differences were observed between sex in the total brain volume, as well as in specific brain regions. For example, significant sex differences were observed in the left (t = 7.525, *p* < 0.001), right cortex (t = 7.556, *p* < 0.001), and total cortex volume (t = 7.564, *p* < 0.001) was larger in males, which points to sex-based variations in the development. Moreover, the analysis revealed significant differences in the cerebral white matter volumes, with both the left (t = 6.943, *p* < 0.001) and right (t = 7.076, *p* < 0.001) hemispheres showing greater WM volumes in males. Total white matter volume (t = 7.021, *p* < 0.001) was also significantly larger in males.

Left (t = 7.169, *p* < 0.001) and right cerebellum cortex (t = 6.415, *p* < 0.001) were showing the same significant sex differences. Larger cerebellum white matter volumes were observed in males, as indicated by the left (t = 3.948, *p* = 0.005) and right cerebellum WM volume (t = 4.029, *p* = 0.004), suggesting greater connectivity and myelination in male brain.

Notably, total subcortical gray matter volume was also significantly larger in males (t = 6.692, *p* < 0.001). The findings related to subcortical regions show sex differences in left (t = 5.393, *p* < 0.001) and right amygdala (t = 6.099, *p* < 0.001), left (t = 5.530, *p* < 0.001) and right thalamus (t = 6.659, *p* < 0.001), left (t = 4.956, *p* < 0.001) and right putamen (t = 5.420, *p* < 0.001), left (t = 5.341, *p* < 0.001) and right pallidum (t = 5.343, *p* < 0.001), left (t = 3.371, *p* = 0.043) and right hippocampus (t = 4.932, *p* < 0.001). Left (t = 5.429, *p* < 0.001) and right ventral DC (t = 6.113, *p* < 0.01) and brain stem showed significant gender differences (t = 6.549, *p* < 0.001) as well. Full details of the results with confidence intervals are in Supplementary Table S4.

These findings are consistent with sex-related differences in the cortical and subcortical regions from other studies, where males exhibit larger total cortical and subcortical volumes, and underscores the potential impact of sex on structures related to complex cognitive functions and emotional regulation (Gennatas et al., 2017; Giedd et al., 1999; Koolschijn and Crone, 2013; Lenroot et al., 2007; Wierenga et al., 2014; Lange et al., 1997).

#### 3.2.2 Cortical thickness

No significant sex differences were observed in regional cortical thickness after correction for multiple comparisons. This finding is consistent with the sex-stratified global mean thickness trajectories reported in section 3.1.4, where modest differences at younger ages largely converged by mid-adolescence without producing systematic regional offsets between males and females (Supplementary Table S5).

#### 3.2.3 Surface area

The analysis revealed statistically significant sex differences in 68 cortical region areas. The strongest statistical significance was observed in the left (t = 2.302, *p* < 0.023) and right (t = 2.54, *p* < 0.012) cortical surface areas, indicating a pronounced effect of sex on overall cortical structure where, boys have larger cortical area. Brain regions that showed significant sex differences are visualized in Figure 5. More details are in Supplementary Table S6.

**Figure 5:**
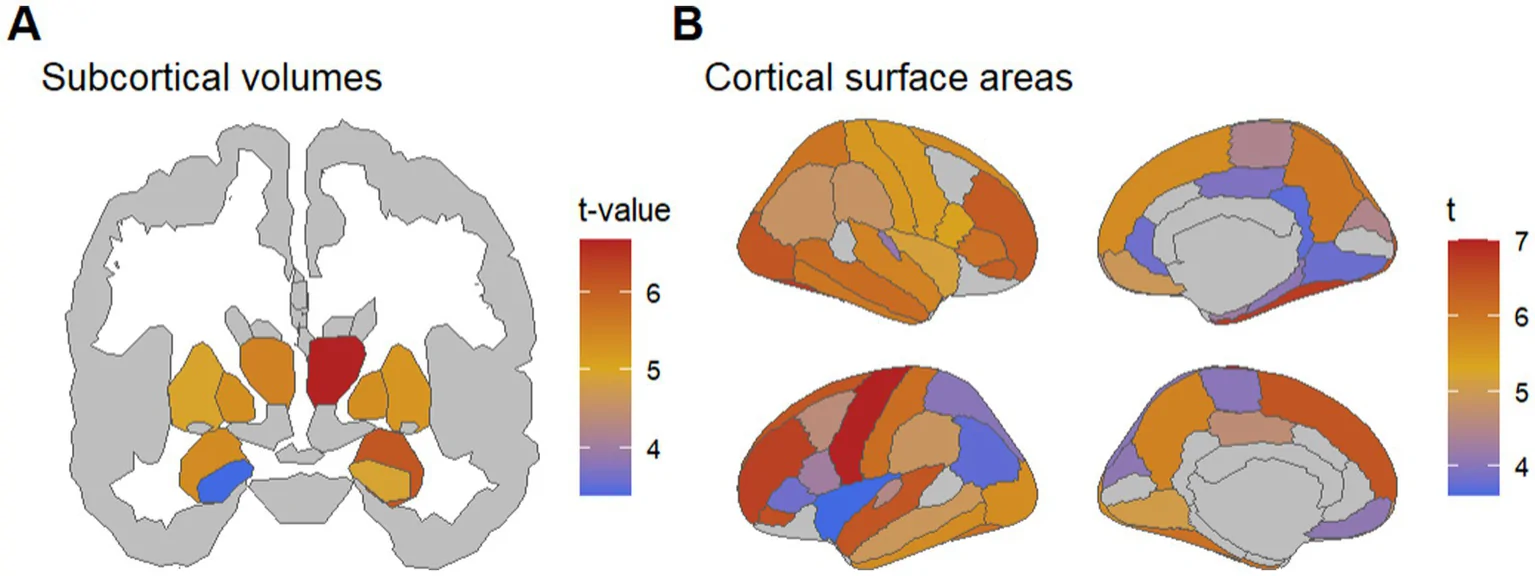
Regional sex differences in subcortical volumes and cortical surface areas. Brain maps display regions showing statistically significant differences between females and males. **(A)** Subcortical structures with significant sex differences in volume. **(B)** Cortical regions with significant sex differences in surface area, shown in lateral and medial views of both hemispheres. Colors represent the corresponding *t*-values, with higher values indicating greater magnitude of sex differences.

### 3.3 Machine learning

#### 3.3.1 Performance leaderboard across feature sets and algorithms

Eleven model/feature-set combinations were evaluated under the same age-stratified five-fold cross-validation scheme (Table 3). The multi-modal stacking ensemble produced the lowest mean absolute error (MAE = 1.55 years, 95% CI [1.38, 1.73], R^2^ = 0.76, Pearson r = 0.87) and the highest fraction of participants predicted within ±1 year (42.5%). Within ±2 years, stacking reached 65.4%, comparable to the best models in Table 3. Among single-modality models, the volumetric features yielded the best performance overall (Lasso on Volume: MAE = 1.67 years, R^2^ = 0.73), followed by Thickness and Area. The Concat matrix generally performed comparably to Volume across algorithms. Conventional R^2^ thresholds for a useful normative brain-age model are approximately R^2^ > 0.6 (Cole and Franke, 2017). All eleven models in Table 3 exceed R^2^ > 0.6, and nine exceed R^2^ > 0.70.

**Table 3:** Out-of-fold performance of all evaluated models.

Model	Feature set	MAE [95% CI]	R^2^ [95% CI]	Pearson r	MedAE	±1 y, %	±2 y, %
Stacking_RidgeMeta	Multi	1.55 [1.38, 1.73]	0.76 [0.71, 0.81]	0.87	1.17	42.5	65.4
LassoCV	Volume	1.67 [1.49, 1.86]	0.73 [0.67, 0.78]	0.85	1.45	32.4	62.6
ElasticNetCV	Concat	1.69 [1.52, 1.89]	0.73 [0.67, 0.77]	0.85	1.44	34.6	67.0
CatBoost_tuned	Concat	1.69 [1.51, 1.86]	0.73 [0.66, 0.78]	0.85	1.35	34.6	65.4
GPR_RBF	Volume	1.71 [1.52, 1.89]	0.72 [0.65, 0.77]	0.85	1.45	34.6	63.7
LightGBM_tuned	Concat	1.71 [1.53, 1.89]	0.71 [0.65, 0.76]	0.84	1.37	39.1	65.4
GPR_RBF	Concat	1.73 [1.55, 1.92]	0.71 [0.65, 0.76]	0.85	1.50	37.4	65.9
XGBoost_tuned	Concat	1.73 [1.55, 1.91]	0.71 [0.64, 0.76]	0.84	1.41	36.3	65.4
LassoCV	Concat	1.74 [1.56, 1.94]	0.70 [0.64, 0.75]	0.84	1.48	36.3	67.0
ElasticNetCV	Volume	1.75 [1.54, 2.00]	0.67 [0.51, 0.76]	0.82	1.47	34.1	62.6
RidgeCV	Concat	1.77 [1.57, 2.02]	0.65 [0.47, 0.76]	0.81	1.51	36.9	66.5

#### 3.3.2 Bias-corrected brain-age estimates

Raw stacking predictions showed the expected regression-to-the-mean bias. The slope of predicted on chronological age was 0.77 and the intercept was +3.16 years. Younger participants were slightly overestimated and older participants were slightly underestimated.

Figure 1 compares raw and bias-corrected out-of-fold predictions from the stacking ensemble. The left panel shows raw predictions. The right panel shows bias-corrected predictions after the procedure of Beheshti et al. (2019). Each point is one participant. Blue points denote male participants and red points denote female participants. The dashed line shows perfect calibration.

The left panel shows the expected deviation from this line across the 7–20 year range. The right panel shows that corrected predictions lie closer to the identity line. This indicates removal of the systematic age-related bias.

Performance also improved after correction. MAE decreased from 1.55 to 1.41 years. R^2^ increased from 0.76 to 0.81. Pearson r increased from 0.87 to 0.92. The proportion of participants predicted within ±2 years increased from 65.4 to 71.5%. These results are summarized in Table 2.

All subsequent sex-stratified analyses and brain-age gap comparisons used the bias-corrected out-of-fold predictions.

MAE decreased from 1.55 to 1.41 years (8.9% reduction), R^2^ increased from 0.76 to 0.81, the Pearson r between predicted and true age increased from 0.87 to 0.92, and the proportion of participants predicted within ±2 years rose from 65.4 to 71.5% (Table 2). All subsequent analyses like sex stratification, brain-age gap testing are based on the bias-corrected predictions.

To check that bias correction removed regression-to-the-mean without new artefacts, we plotted mean prediction error in one-year age bins in Figure 6. Raw predictions were positive in younger bins and negative in older bins. After Beheshti correction the bin-wise means clustered near zero across the 7–20 year range. The corrected line did not show a systematic age slope.

**Figure 6:**
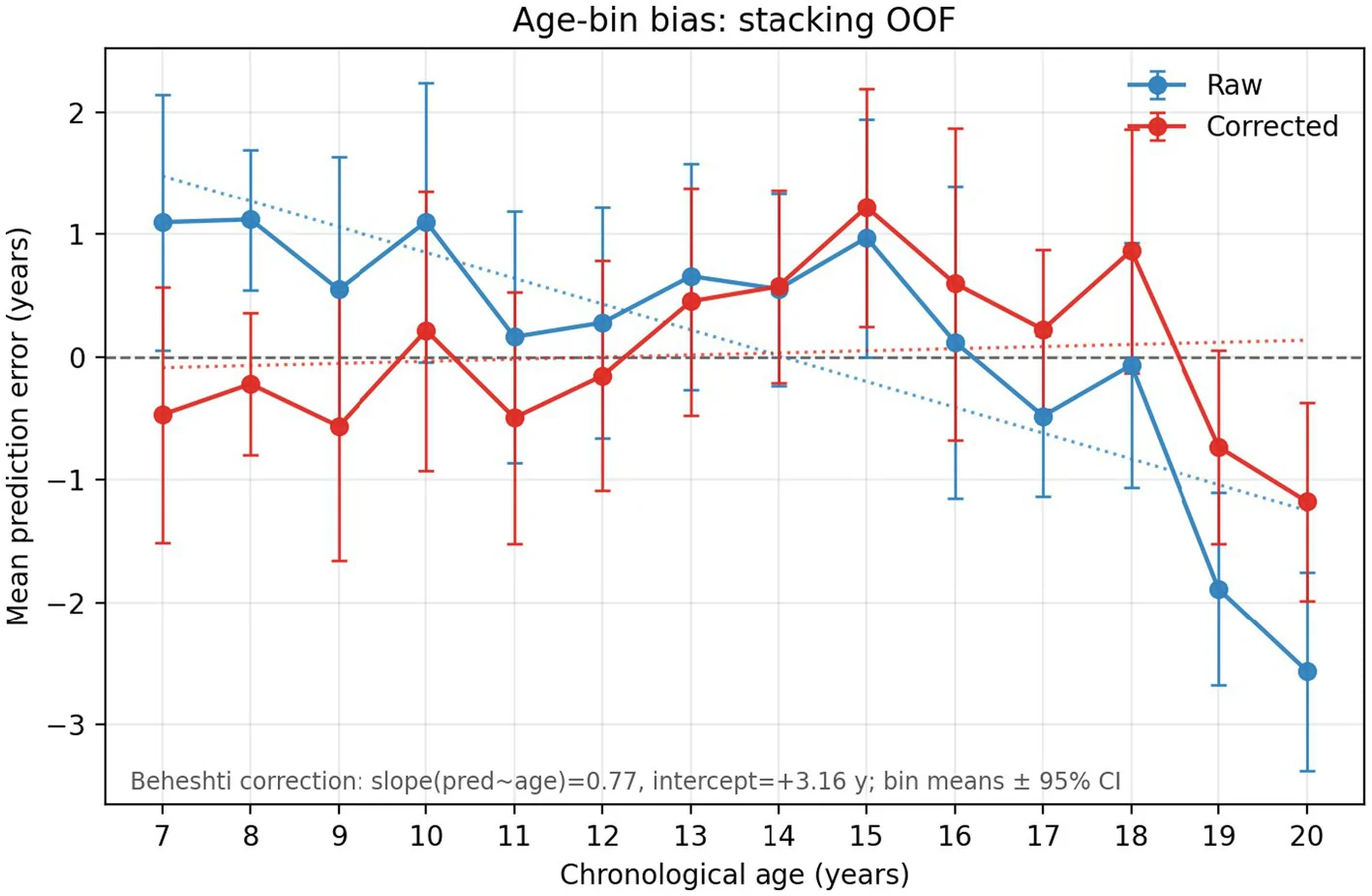
Age-bin prediction bias. Mean out-of-fold prediction error is shown for one-year age bins, with error bars indicating 95% confidence intervals. Blue shows raw predictions and red shows bias-corrected predictions, with the dashed horizontal line at zero indicating no bias. Dotted lines show linear trends across age bins, illustrating regression to the mean in raw predictions and its reduction after Beheshti correction.

#### 3.3.3 Sex-stratified analysis

Two complementary sex-stratified designs were analysed, as shown in Table 4. In Design A, a single stacking model was trained on the full sample and its out-of-fold predictions were evaluated separately in 88 females and 91 males. MAE was nearly identical between sexes. On raw predictions it was 1.53 years in females and 1.57 years in males. After correction it was 1.39 and 1.43 years. The corrected brain-age gap also did not differ significantly. The mean corrected gap was +0.03 years in males and −0.03 years in females. The Mann–Whitney U test gave U = 4,109 and *p* = 0.76. Design A is therefore the main confirmatory test of sex-related bias in brain-age estimation. All brain-age gap comparisons by sex used bias-corrected out-of-fold predictions.

**Table 4:** Sex-stratified machine-learning performance.

Design	Model	Sex	*n*	MAE, y	R^2^	Pearson r
A - shared model	Stacking_RidgeMeta	Female	88	1.53	0.77	0.88
A - shared model	Stacking_RidgeMeta	Male	91	1.57	0.75	0.87
B - sex-specific	Stacking_RidgeMeta	Female	88	1.46	0.78	0.88
B - sex-specific	Stacking_RidgeMeta	Male	91	1.72	0.71	0.84

In Design B, separate stacking models were trained within each sex. MAE was numerically lower in males than in females, 1.46 versus 1.72 years. This comparison is underpowered because each sex-specific model was trained on only about 88 to 91 participants while still using the full feature set of about 151 morphometric measures. The difference between Designs A and B should therefore be treated as exploratory. It should not be interpreted as evidence of a stable sex difference in predictability. Larger samples are needed to test this question.

#### 3.3.4 Feature importance and anatomical interpretation

Two independent interpretability analyses converged on a consistent anatomical story (Table 5). The top SHAP features of the tuned CatBoost model on the Concat matrix were dominated by bilateral cortical thickness measurements. Left and right precuneus thickness (mean |SHAP| = 0.44 and 0.41, respectively), followed by left entorhinal cortex thickness, right inferior parietal thickness and left rostral anterior cingulate thickness. The Lasso ranking on the full sample retained the same regions but additionally selected several volumetric features. Right cerebral white-matter volume, brainstem volume and the central segment of the corpus callosum with positive coefficients, indicating that these volumes tend to be larger in older adolescents, consistent with continuing myelination. Sex-specific Lasso models showed preliminary overlap but also suggestive divergences. In males, the largest absolute coefficients were observed for the central corpus callosum and brainstem volumes. In females, greater weight was placed on right nucleus accumbens volume and left lingual thickness. These models were fit separately on about 88 to 91 participants per sex. The sex-specific rankings are therefore exploratory. They may reflect sampling variability rather than stable sex differences and should not be interpreted as confirmatory evidence for distinct maturation trajectories. The pooled-sample SHAP and Lasso results remain the main basis for anatomical interpretation.

**Table 5:** Top-10 features by SHAP and Lasso ranking.

#	SHAP top region	|SHAP|	Lasso (all) region	coef	Lasso (male) region	coef	Lasso (female) region	coef
1	L_precuneus_thick (thick)	0.44	rhCerebralWhiteMatterVol (vol)	+0.63	CC_Central (vol)	+0.73	Right_Accumbens_area (vol)	−0.53
2	R_precuneus_thick (thick)	0.41	L_entorhinal_thick (thick)	+0.45	Brain_Stem (vol)	+0.68	L_lingual_thick (thick)	−0.48
3	L_entorhinal_thick (thick)	0.31	Brain_Stem (vol)	+0.42	R_precuneus_thick (thick)	−0.62	L_entorhinal_thick (thick)	+0.37
4	R_inferiorparietal_thick (thick)	0.25	L_superiorparietal_thick (thick)	−0.38	L_lateralorbitofrontal_thick (thick)	−0.61	L_parstriangularis_thick (thick)	−0.37
5	L_rostralanteriorcingulate_thick (thick)	0.25	third_Ventricle (vol)	+0.34	L_isthmuscingulate_thick (thick)	−0.59	L_parsorbitalis_thick (thick)	−0.36
6	R_superiorparietal_thick (thick)	0.23	L_precuneus_thick (thick)	−0.33	R_lateraloccipital (area)	−0.47	L_precuneus_thick (thick)	−0.36
7	L_isthmuscingulate_thick (thick)	0.22	CC_Central (vol)	+0.32	L_transversetemporal_thick (thick)	−0.43	third_Ventricle (vol)	+0.35
8	R_parahippocampal_thick (thick)	0.20	L_lateralorbitofrontal_thick (thick)	−0.32	L_superiorparietal_thick (thick)	−0.38	R_frontalpole_thick (thick)	−0.29
9	R_rostralmiddlefrontal_thick (thick)	0.18	L_transversetemporal_thick (thick)	−0.32	L_inferiorparietal_thick (thick)	−0.31	L_superiorparietal_thick (thick)	−0.28
10	R_precentral_thick (thick)	0.17	L_middletemporal_thick (thick)	−0.30	third_Ventricle (vol)	+0.24	R_bankssts_thick (thick)	−0.28

## 4 Discussion

In the present study, we demonstrated the robust associations between participants’ age and brain volume, aligned broadly with neuroanatomical maturation from 7 to 20 years. We showed significant negative correlations between age and cortical volume across both hemispheres as well as total gray matter, supporting the well-documented trend of gray-matter volume reduction.

Interestingly, age correlated positively with cerebral and cerebellar white matter volume. Such findings are in line with developmental trajectories where white matter increases during childhood and early adulthood (Bethlehem et al., 2022; Koppelmans et al., 2015).

The negative correlations established between age and volumes of the right caudate nucleus and bilateral nucleus accumbens are consonant with cross-sectional findings demonstrating age-related shrinkage in these regions (Walhovd et al., 2011).

The present study also identified widespread age-related changes in cortical thickness across multiple brain regions, with the majority showing significant negative correlations with age. These findings align with previous neuroimaging studies demonstrating progressive cortical thinning from childhood through adolescence (Shaw et al., 2008; Tamnes et al., 2010). Such thinning is a reflection of synaptic pruning, increased myelination, and refinement of neural circuits to support more efficient cognitive processing (Sowell et al., 2004).

Both left and right superior temporal sulcus exhibited strong negative correlations with age. This region plays a key role in social perception and language processing, and its age-related thinning reflects developmental specialization in higher-order auditory and language functions (Deen et al., 2015).

The caudal and rostral middle frontal cortex, components of the prefrontal cortex, also demonstrated thinning with age. This finding is consistent with the extended developmental trajectory of the prefrontal cortex, which underpins executive function, planning, and decision-making (Casey et al., 2005; Lenroot et al., 2007). Notably, the rostral middle frontal cortex displayed some of the strongest correlations observed, suggesting that protracted maturation in this region is particularly pronounced during the studied period. Significant negative correlations were also found with cuneus, a visual processing area. This observation mirrors prior evidence that visual cortices undergo measurable structural changes during youth, reflecting fine-tuning of visual perception and visuospatial integration (Brown et al., 2012). The observed cortical thinning in these regions indicates the brain’s shift towards more efficient and specialized neural networks. In language-related cortices, this may enhance semantic and syntactic processing efficiency. In executive areas, such as the prefrontal cortex and ACC, structural maturation supports improved working memory, inhibitory control, and self-regulation (Tamnes et al., 2017). Our results are consistent with developmental neuroimaging findings that highlight a posterior-to-anterior gradient of cortical thinning, with sensory areas maturing earlier than association cortices (Gogtay et al., 2004). Moreover, the bilateral symmetry in thinning patterns across frontal and temporal cortices reinforces the notion that these maturational processes are coordinated across hemispheres (Shaw et al., 2008).

The current study revealed pronounced sex-related differences in brain morphology, with males demonstrating larger total brain volumes, as well as larger volumes in multiple cortical, subcortical, cerebellar, and white matter regions. These differences persisted after controlling for age and intracranial volume, consistent with prior neuroimaging studies showing global volumetric advantages in males (Ruigrok et al., 2014).

No significant sex differences were observed in cortical thickness across regions, indicating that male–female differences in brain structure are primarily driven by surface area and overall volume rather than thickness. This finding is in line with prior reports that cortical thickness is relatively similar across sexes when controlling for brain size (Luders et al., 2006; Sowell et al., 2007).

One of the purpose of this study was the strong body of literature suggesting that females generally exhibit earlier cortical maturation than males during childhood and adolescence (Tamnes et al., 2017; Crone and Dahl, 2012; Gennatas et al., 2017; Koolschijn and Crone, 2013; Lenroot et al., 2007). Contrary to this expectation, we did not find significant sex differences in cortical thickness after correction for multiple comparisons, nor did we observe differences in the corrected brain-age gap between boys and girls. These findings suggest that, although sex-related differences in neurodevelopmental trajectories are well established, they may not be necessarily connected with observable differences in global brain-age estimates within a healthy pediatric population.

Several factors may explain this probable inconsistency. First, previous studies that demonstrate earlier female maturation have mainly characterized developmental trajectories at the regional level rather than differences in overall maturational status at a single time point. Longitudinal neuroimaging studies have shown that girls generally reach peaks of cortical thickness and subsequent cortical thinning earlier than boys, whereas the overall sequence of cortical maturation is mostly similar between sexes (Giedd et al., 1999; Lenroot et al., 2007). Consequently, boys and girls may follow temporally shifted developmental trajectories that nevertheless overlap considerably across the age range examined in the present study. Such overlap would reduce the likelihood of detecting significant sex differences in cross-sectional analyses.

Second, brain-age models are designed to outline information distributed across the whole brain rather than quantify maturation within individual cortical regions. Sex differences described in previous structural MRI studies are often region-specific and vary considerably across cortical areas (Gennatas et al., 2017; Tamnes et al., 2017). When these heterogeneous regional effects are integrated into a single prediction model, opposite or compensatory developmental patterns across different brain regions may weaken sex-specific differences in the resulting brain-age estimate. Therefore, the absence of sex differences in the corrected brain-age gap does not necessarily demonstrate identical neurodevelopmental processes but rather suggests that global maturational status is comparable between healthy boys and girls after accounting for chronological age.

Another consideration is that sex differences in cortical development are generally characterized by modest effect sizes and substantial inter-individual variability (Mills et al., 2016; Tamnes et al., 2017). Increasing evidence suggests that variability within each sex exceeds average differences between sexes for many neuroanatomical measures (Joel et al., 2015). Consequently, machine-learning models trained to predict chronological age may depend mostly on age-related anatomical features that are shared across individuals, whereas relatively subtle sex-related variation contributes little additional predictive information.

Our findings are also consistent with previous brain-age studies reporting limited or absent sex differences after age correction. While sex-specific developmental trajectories undoubtedly exist, several investigations have shown that brain-age prediction models generalize similarly across males and females and that the resulting brain-age gap is often not significantly associated with biological sex in healthy populations (Franke and Gaser, 2019; Kaufmann et al., 2019). This suggests that chronological age is the dominant determinant of structural brain-age estimates, whereas sex-related anatomical differences contribute relatively little to overall prediction error.

In contrast, widespread sex-related differences were found in cortical surface area, with males exhibiting significantly larger areas in cortical regions. The largest effects were observed for the left and right total cortical surface areas, consistent with evidence that surface area contributes more to overall brain size differences between sexes than cortical thickness (Fjell et al., 2013).

Having characterised age- and sex-related morphometric patterns in our cohort, we next evaluated whether multivariate combinations of these structural features could predict chronological age. The multi-modal stacking ensemble predicted chronological age with a bias-corrected mean absolute error of 1.41 years and explained 81% of the variance in our 7-20-year-old sample. This level of accuracy is competitive with the best published estimates on adolescent cohorts of comparable age range and sample size: Khundrakpam et al. (2015) reported a mean absolute error of 1.0–1.8 years on the multi-site NIH-PD cohort using cortical-thickness alone, and Ball et al. (2021) reported individual variation around an MAE of approximately 1.5–2 years on typically developing children. Larger adult studies routinely report higher absolute errors (Cole and Franke, 2017; Bashyam et al., 2020), reflecting both a wider chronological span and the slower structural changes of adulthood. That a comparatively small, single-site sample suffices to reach this accuracy suggests that adolescent brain maturation leaves a strong, multivariate signature.

Regarding anatomical convergence between linear and non-linear models, the SHAP analysis of CatBoost and the sparse coefficients of Lasso identified an overlapping set of features as the dominant predictors of age, such as bilateral precuneus thickness, left entorhinal cortex thickness, right inferior parietal thickness, the central segment of the corpus callosum and right cerebral white-matter volume. The convergence of an L1-regularised linear model and a non-linear gradient-boosting model on the same regions indicates that the bulk of the age signal is carried by largely linear changes in cortical thickness and white-matter volume. This finding is consistent with the broader developmental literature on synaptic pruning and myelination (Shaw et al., 2008; Tamnes et al., 2017). The incremental advantage of gradient boosting and the stacking ensemble probably reflects subtle interactions among these features rather than fundamentally different anatomical substrates.

Although the volumetric and surface-area analyses in section 3.2 revealed pronounced sex differences in absolute brain size, these differences did not translate into a sex bias in the brain-age predictor when the model was trained on the combined sample. The corrected brain-age gap was statistically indistinguishable between males and females (+0.03 y versus −0.03 y, Mann–Whitney U = 4,109, *p* = 0.76). Taken together, this suggests that the model reflects developmental stage rather than overall brain size. When separate models were trained within each sex in Design B, performance was numerically lower and sex differences appeared larger than in Design A. This design is underpowered with 179 participants and should be viewed as exploratory. These findings mainly support pooling sexes for age prediction. Sex-stratified modelling is better reserved for larger validation samples.

Together, the morphometric and brain-age analyses describe a coherent picture of adolescent brain maturation in this cohort. Gray-matter volumes declined and white-matter volumes increased with age, cortical thinning was widespread, sex differences were most evident in brain scale rather than thickness, and multivariate structural features nonetheless predicted chronological age with useful accuracy. These results provide a preliminary description of brain maturation in this Central Asian adolescent cohort. The brain-age models require external validation before use as a normative reference.

## 5 Limitations

Several limitations should be considered when interpreting the present findings. First, the study applied a cross-sectional design, which precludes direct assessment of within-individual neurodevelopmental trajectories. Although significant age-related associations were found, these findings should not be interpreted as evidence of causal developmental processes or maturational change over time. Longitudinal studies are required to distinguish true developmental trajectories from between-individual variability.

Second, participants were recruited from a single imaging centre and represented a relatively homogeneous population. Consequently, the present findings characterize structural brain development within this cohort and should not be interpreted as universally applicable normative developmental trajectories. The generalizability of these results to populations with different genetic, environmental, demographic, or socioeconomic characteristics remains to be studied through multicentre studies involving more diverse cohorts.

The sample size of 179 participants is modest for high-dimensional brain-age modelling and limits sex-stratified inferences. Each participant was described by about 151 morphometric features. We therefore used regularized models, age-stratified repeated five-fold cross-validation, bootstrap confidence intervals for MAE and R^2^ in Table 3, and Wilcoxon comparisons between models. These steps support the main pooled stacking model but do not provide formal power for every secondary comparison.

We separate confirmatory and exploratory machine-learning analyses. In Design A, one model was trained on the full sample and evaluated separately by sex. This analysis is the main basis for concluding that the corrected brain-age gap did not differ between females and males. In Design B, separate models were trained within each sex on about 88 to 91 participants. The sex-specific Lasso rankings should be treated the same way. These secondary analyses are exploratory and hypothesis-generating rather than confirmatory. With fewer than 100 cases per sex and many input features, their results may reflect sampling variability and should not be overinterpreted.

Further limitations include the single-site ethnically homogeneous cohort and the lack of independent external validation, which limit generalizability. Small-sample bias was additionally reduced through regularized model selection and Beheshti bias-correction.

Finally, although brain-age prediction provides a useful summary measure of structural brain maturation, it represents a global estimate derived from multiple neuroanatomical features and may not capture subtle regional developmental differences. Therefore, the absence of significant sex differences in the corrected brain-age gap should not be interpreted as evidence that regional developmental trajectories are identical between males and females.

Despite these limitations, the study provides valuable structural MRI data from a healthy paediatric cohort that is currently underrepresented in the neuroimaging literature and contributes to expanding normative reference data for future developmental neuroimaging research.

## Data Availability

The datasets presented in this article are not readily available because restricted by Ethics Committee of al-Farabi Kazakh National University. Requests to access the datasets should be directed to Kustubayeva Almira, almkusto@kaznu.kz.

## Footnotes

1
https://surfer.nmr.mgh.harvard.edu
