# Supplementary material for: Age- and sex-divergent neurodevelopmental patterns revealed through structural magnetic resonance imaging features
Source: Front Neuroanat. 2026 Sep 21;20:1906618. doi: 10.3389/fnana.2026.1906618 (PMC13635291; doi:10.3389/fnana.2026.1906618)
Supplement: Supplementary file 1 [file Table_1.docx]

Supplementary material.

Table S1. A) Raw cortical volume correlation with age.

| **Variable** | **N** | **r** | **CI_lower** | **CI_upper** | **p_raw** | **p_adj** |
| --- | --- | --- | --- | --- | --- | --- |
| lhCortexVol | 179 | −0.504 | −0.606 | −0.385 | **0.000** | **0.000** |
| rhCortexVol | 179 | −0.493 | −0.596 | −0.373 | **0.000** | **0.000** |
| CortexVol | 179 | −0.499 | −0.602 | −0.380 | **0.000** | **0.000** |
| lhCerebralWhiteMatterVol | 179 | 0.309 | 0.17 | 0.436 | **0.000** | **0.001** |
| rhCerebralWhiteMatterVol | 179 | 0.316 | 0.178 | 0.443 | **0.000** | **0.001** |
| CerebralWhiteMatterVol | 179 | 0.313 | 0.175 | 0.440 | **0.000** | **0.001** |
| SubCortGrayVol | 179 | −0.045 | −0.190 | 0.103 | 0.553 | 1.000 |
| Left_Lateral_Ventricle | 179 | −0.048 | −0.193 | 0.099 | 0.523 | 1.000 |
| Left_Inf_Lat_Vent | 179 | 0.096 | −0.051 | 0.239 | 0.201 | 1.000 |
| Left_Cerebellum_White_Matter | 179 | 0.309 | 0.17 | 0.436 | **0.000** | **0.001** |
| Left_Cerebellum_Cortex | 179 | 0.032 | −0.115 | 0.178 | 0.669 | 1.000 |
| **Left_Thalamus** | 179 | 0.046 | −0.101 | 0.192 | 0.538 | 1.000 |
| **Left_Caudate** | 179 | −0.169 | −0.308 | −0.023 | **0.024** | 1.000 |
| **Left_Putamen** | 179 | −0.161 | −0.301 | −0.015 | **0.031** | 1.000 |
| **Left_Pallidum** | 179 | 0.141 | −0.006 | 0.281 | 0.061 | 1.000 |
| third_Ventricle | 179 | 0.332 | 0.194 | 0.456 | **0.000** | **0.000** |
| forth_Ventricle | 179 | 0.071 | −0.077 | 0.215 | 0.348 | 1.000 |
| **Brain_Stem** | 179 | 0.445 | 0.319 | 0.555 | **0.000** | **0.000** |
| **Left_Hippocampus** | 179 | 0.104 | −0.043 | 0.247 | 0.166 | 1.000 |
| **Left_Amygdala** | 179 | −0.209 | −0.346 | −0.065 | **0.005** | 0.230 |
| CSF | 179 | 0.227 | 0.083 | 0.361 | **0.002** | 0.107 |
| **Left_Accumbens_area** | 179 | −0.397 | −0.514 | −0.266 | **0.000** | **0.000** |
| **Left_VentralDC** | 179 | 0.368 | 0.234 | 0.488 | **0.000** | **0.000** |
| Left_choroid_plexus | 179 | 0.058 | −0.089 | 0.203 | 0.440 | 1.000 |
| Right_Lateral_Ventricle | 179 | 0.079 | −0.068 | 0.223 | 0.291 | 1.000 |
| Right_Inf_Lat_Vent | 179 | 0.06 | −0.087 | 0.205 | 0.423 | 1.000 |
| Right_Cerebellum_White_Matter | 179 | 0.311 | 0.173 | 0.438 | 0.000 | **0.001** |
| Right_Cerebellum_Cortex | 179 | 0.059 | −0.088 | 0.204 | 0.433 | 1.000 |
| **Right_Thalamus** | 179 | 0.021 | −0.127 | 0.167 | 0.785 | 1.000 |
| **Right_Caudate** | 179 | −0.247 | −0.380 | −0.104 | **0.001** | **0.040** |
| **Right_Putamen** | 179 | −0.142 | −0.283 | 0.005 | 0.058 | 1.000 |
| **Right_Pallidum** | 179 | 0.216 | 0.072 | 0.352 | **0.004** | 0.170 |
| **Right_Hippocampus** | 179 | 0.089 | −0.058 | 0.233 | 0.236 | 1.000 |
| **Right_Amygdala** | 179 | −0.096 | −0.240 | 0.051 | 0.200 | 1.000 |
| **Right_Accumbens_area** | 179 | −0.424 | −0.537 | −0.295 | **0.000** | **0.000** |
| **Right_VentralDC** | 179 | 0.311 | 0.172 | 0.438 | **0.000** | **0.001** |
| Right_choroid_plexus | 179 | 0.03 | −0.118 | 0.175 | 0.694 | 1.000 |
| WM_hypointensities | 179 | −0.228 | −0.363 | −0.084 | **0.002** | 0.099 |
| Optic_Chiasm | 179 | 0.031 | −0.116 | 0.177 | 0.678 | 1.000 |
| CC_Posterior | 179 | 0.219 | 0.075 | 0.355 | **0.003** | 0.149 |
| CC_Mid_Posterior | 179 | 0.128 | −0.019 | 0.270 | 0.087 | 1.000 |
| CC_Central | 179 | 0.299 | 0.159 | 0.427 | **0.000** | **0.002** |
| CC_Mid_Anterior | 179 | 0.239 | 0.096 | 0.373 | **0.001** | 0.060 |

Table S1. B) Scaled by total ICV cortical volume correlation with age.

| **Variable** | **N** | **r** | **CI_lower** | **CI_upper** | **p_raw** | **p_adj** |
| --- | --- | --- | --- | --- | --- | --- |
| lhCortexVol | 174 | −0.309 | −0.438 | −0.168 | 0.000 | **0.002** |
| rhCortexVol | 174 | −0.299 | −0.429 | −0.158 | 0.000 | **0.003** |
| CortexVol | 174 | −0.305 | −0.434 | −0.163 | 0.000 | **0.002** |
| lhCerebralWhiteMatterVol | 174 | 0.33 | 0.191 | 0.456 | 0.000 | **0.000** |
| rhCerebralWhiteMatterVol | 174 | 0.334 | 0.195 | 0.46 | 0.000 | **0.000** |
| CerebralWhiteMatterVol | 174 | 0.332 | 0.193 | 0.458 | 0.000 | **0.000** |
| SubCortGrayVol | 174 | 0.07 | −0.080 | 0.217 | 0.358 | 1.000 |
| Left_Lateral_Ventricle | 174 | −0.048 | −0.195 | 0.102 | 0.532 | 1.000 |
| Left_Inf_Lat_Vent | 174 | 0.118 | −0.032 | 0.262 | 0.122 | 1.000 |
| Left_Cerebellum_White_Matter | 174 | 0.308 | 0.167 | 0.437 | 0.000 | **0.002** |
| Left_Cerebellum_Cortex | 174 | 0.115 | −0.034 | 0.26 | 0.130 | 1.000 |
| Left_Thalamus | 174 | 0.128 | −0.021 | 0.272 | 0.092 | 1.000 |
| Left_Caudate | 174 | −0.058 | −0.205 | 0.092 | 0.447 | 1.000 |
| Left_Putamen | 174 | −0.040 | −0.187 | 0.11 | 0.603 | 1.000 |
| Left_Pallidum | 174 | 0.176 | 0.028 | 0.316 | 0.021 | 0.962 |
| third_Ventricle | 174 | 0.342 | 0.204 | 0.467 | 0.000 | **0.000** |
| forth_Ventricle | 174 | 0.099 | −0.050 | 0.245 | 0.192 | 1.000 |
| Brain_Stem | 174 | 0.411 | 0.279 | 0.527 | 0.000 | **0.000** |
| Left_Hippocampus | 174 | 0.169 | 0.021 | 0.31 | 0.026 | 1.000 |
| Left_Amygdala | 174 | −0.080 | −0.226 | 0.069 | 0.293 | 1.000 |
| CSF | 174 | 0.245 | 0.099 | 0.38 | 0.001 | 0.054 |
| Left_Accumbens_area | 174 | −0.273 | −0.406 | −0.130 | 0.000 | **0.012** |
| Left_VentralDC | 174 | 0.337 | 0.198 | 0.463 | 0.000 | **0.000** |
| Left_choroid_plexus | 174 | 0.08 | −0.070 | 0.226 | 0.296 | 1.000 |
| Right_Lateral_Ventricle | 174 | 0.096 | −0.053 | 0.242 | 0.206 | 1.000 |
| Right_Inf_Lat_Vent | 174 | 0.096 | −0.053 | 0.242 | 0.206 | 1.000 |
| Right_Cerebellum_White_Matter | 174 | 0.308 | 0.167 | 0.437 | 0.000 | **0.002** |
| Right_Cerebellum_Cortex | 174 | 0.129 | −0.020 | 0.272 | 0.091 | 1.000 |
| Right_Thalamus | 174 | 0.113 | −0.037 | 0.257 | 0.139 | 1.000 |
| Right_Caudate | 174 | −0.120 | −0.264 | 0.029 | 0.115 | 1.000 |
| Right_Putamen | 174 | −0.021 | −0.169 | 0.128 | 0.786 | 1.000 |
| Right_Pallidum | 174 | 0.231 | 0.085 | 0.367 | 0.002 | 0.102 |
| Right_Hippocampus | 174 | 0.16 | 0.011 | 0.301 | 0.035 | 1.000 |
| Right_Amygdala | 174 | 0.01 | −0.139 | 0.158 | 0.896 | 1.000 |
| Right_Accumbens_area | 174 | −0.288 | −0.419 | −0.146 | 0.000 | **0.005** |
| Right_VentralDC | 174 | 0.304 | 0.163 | 0.433 | 0.000 | **0.002** |
| Right_choroid_plexus | 174 | 0.061 | −0.089 | 0.208 | 0.423 | 1.000 |
| WM_hypointensities | 174 | −0.208 | −0.346 | −0.061 | 0.006 | 0.282 |
| Optic_Chiasm | 174 | 0.087 | −0.062 | 0.233 | 0.252 | 1.000 |
| CC_Posterior | 174 | 0.233 | 0.087 | 0.369 | 0.002 | 0.093 |
| CC_Mid_Posterior | 174 | 0.161 | 0.012 | 0.302 | 0.034 | 1.000 |
| CC_Central | 174 | 0.332 | 0.192 | 0.458 | 0.000 | **0.000** |
| CC_Mid_Anterior | 174 | 0.282 | 0.139 | 0.413 | 0.000 | **0.008** |

Table S2. Raw surface area correlated with age.

| **Variable** | **N** | **r** | **CI_lower** | **CI_upper** | **p_raw** | **p_adj** |
| --- | --- | --- | --- | --- | --- | --- |
| L_inferiorparietal | 179 | −0.224 | −0.359 | −0.080 | **0.003** | 0.173 |
| R_superiorparietal | 179 | −0.219 | −0.354 | −0.075 | **0.003** | 0.218 |
| L_bankssts | 179 | −0.046 | −0.191 | 0.101 | 0.540 | 1.000 |
| L_caudalanteriorcingulate | 179 | −0.069 | −0.213 | 0.079 | 0.359 | 1.000 |
| L_caudalmiddlefrontal | 179 | −0.016 | −0.162 | 0.131 | 0.832 | 1.000 |
| L_cuneus | 179 | 0.001 | −0.145 | 0.148 | 0.985 | 1.000 |
| L_entorhinal | 179 | 0.000 | −0.147 | 0.146 | 0.998 | 1.000 |
| L_fusiform | 179 | −0.017 | −0.163 | 0.130 | 0.826 | 1.000 |
| L_inferiortemporal | 179 | 0.011 | −0.136 | 0.157 | 0.886 | 1.000 |
| L_isthmuscingulate | 179 | −0.135 | −0.277 | 0.012 | 0.071 | 1.000 |
| L_lateraloccipital | 179 | −0.061 | −0.206 | 0.087 | 0.419 | 1.000 |
| L_lateralorbitofrontal | 179 | −0.025 | −0.171 | 0.122 | 0.736 | 1.000 |
| L_lingual | 179 | −0.040 | −0.185 | 0.108 | 0.600 | 1.000 |
| L_medialorbitofrontal | 179 | 0.026 | −0.121 | 0.172 | 0.729 | 1.000 |
| L_middletemporal | 179 | −0.045 | −0.190 | 0.102 | 0.550 | 1.000 |
| L_parahippocampal | 179 | 0.026 | −0.121 | 0.172 | 0.732 | 1.000 |
| L_paracentral | 179 | −0.129 | −0.271 | 0.018 | 0.084 | 1.000 |
| L_parsopercularis | 179 | −0.072 | −0.216 | 0.076 | 0.339 | 1.000 |
| L_parsorbitalis | 179 | −0.039 | −0.184 | 0.109 | 0.607 | 1.000 |
| L_parstriangularis | 179 | −0.120 | −0.262 | 0.028 | 0.111 | 1.000 |
| L_pericalcarine | 179 | 0.071 | −0.076 | 0.216 | 0.344 | 1.000 |
| L_postcentral | 179 | −0.128 | −0.269 | 0.019 | 0.088 | 1.000 |
| L_posteriorcingulate | 179 | −0.115 | −0.257 | 0.032 | 0.125 | 1.000 |
| L_precentral | 179 | −0.056 | −0.201 | 0.092 | 0.458 | 1.000 |
| L_precuneus | 179 | −0.106 | −0.249 | 0.041 | 0.157 | 1.000 |
| L_rostralanteriorcingulate | 179 | 0.023 | −0.124 | 0.169 | 0.762 | 1.000 |
| L_rostralmiddlefrontal | 179 | −0.048 | −0.194 | 0.099 | 0.520 | 1.000 |
| L_superiorfrontal | 179 | −0.068 | −0.212 | 0.080 | 0.366 | 1.000 |
| L_superiorparietal | 179 | −0.099 | −0.242 | 0.049 | 0.190 | 1.000 |
| L_superiortemporal | 179 | −0.039 | −0.185 | 0.108 | 0.602 | 1.000 |
| L_supramarginal | 179 | −0.029 | −0.175 | 0.118 | 0.699 | 1.000 |
| L_frontalpole | 179 | −0.110 | −0.253 | 0.037 | 0.142 | 1.000 |
| L_temporalpole | 179 | 0.008 | −0.139 | 0.155 | 0.915 | 1.000 |
| L_transversetemporal | 179 | −0.058 | −0.203 | 0.090 | 0.441 | 1.000 |
| L_insula | 179 | −0.040 | −0.185 | 0.107 | 0.596 | 1.000 |
| R_bankssts | 179 | −0.033 | −0.179 | 0.114 | 0.663 | 1.000 |
| R_caudalanteriorcingulate | 179 | 0.006 | −0.141 | 0.153 | 0.935 | 1.000 |
| R_caudalmiddlefrontal | 179 | −0.117 | −0.259 | 0.030 | 0.118 | 1.000 |
| R_cuneus | 179 | −0.049 | −0.195 | 0.098 | 0.512 | 1.000 |
| R_entorhinal | 179 | 0.017 | −0.130 | 0.164 | 0.818 | 1.000 |
| R_fusiform | 179 | −0.047 | −0.192 | 0.101 | 0.535 | 1.000 |
| R_inferiorparietal | 179 | −0.105 | −0.248 | 0.042 | 0.160 | 1.000 |
| R_inferiortemporal | 179 | 0.096 | −0.051 | 0.239 | 0.202 | 1.000 |
| R_isthmuscingulate | 179 | −0.003 | −0.149 | 0.144 | 0.970 | 1.000 |
| R_lateraloccipital | 179 | −0.072 | −0.216 | 0.076 | 0.341 | 1.000 |
| R_lateralorbitofrontal | 179 | 0.010 | −0.137 | 0.156 | 0.894 | 1.000 |
| R_lingual | 179 | −0.061 | −0.206 | 0.087 | 0.419 | 1.000 |
| R_medialorbitofrontal | 179 | −0.041 | −0.186 | 0.107 | 0.589 | 1.000 |
| R_middletemporal | 179 | −0.012 | −0.158 | 0.135 | 0.875 | 1.000 |
| R_parahippocampal | 179 | −0.114 | −0.256 | 0.033 | 0.129 | 1.000 |
| R_paracentral | 179 | −0.115 | −0.257 | 0.032 | 0.126 | 1.000 |
| R_parsopercularis | 179 | −0.062 | −0.207 | 0.086 | 0.411 | 1.000 |
| R_parsorbitalis | 179 | −0.056 | −0.201 | 0.092 | 0.458 | 1.000 |
| R_parstriangularis | 179 | −0.081 | −0.225 | 0.066 | 0.281 | 1.000 |
| R_pericalcarine | 179 | 0.039 | −0.108 | 0.185 | 0.601 | 1.000 |
| R_postcentral | 179 | −0.134 | −0.275 | 0.013 | 0.074 | 1.000 |
| R_posteriorcingulate | 179 | −0.075 | −0.219 | 0.073 | 0.319 | 1.000 |
| R_precentral | 179 | −0.069 | −0.213 | 0.079 | 0.361 | 1.000 |
| R_precuneus | 179 | −0.141 | −0.281 | 0.006 | 0.061 | 1.000 |
| R_rostralanteriorcingulate | 179 | −0.038 | −0.184 | 0.109 | 0.610 | 1.000 |
| R_rostralmiddlefrontal | 179 | −0.031 | −0.177 | 0.116 | 0.680 | 1.000 |
| R_superiorfrontal | 179 | −0.052 | −0.197 | 0.096 | 0.494 | 1.000 |
| R_superiortemporal | 179 | −0.003 | −0.150 | 0.144 | 0.967 | 1.000 |
| R_supramarginal | 179 | −0.034 | −0.180 | 0.113 | 0.649 | 1.000 |
| R_frontalpole | 179 | −0.081 | −0.225 | 0.067 | 0.282 | 1.000 |
| R_temporalpole | 179 | 0.020 | −0.127 | 0.166 | 0.790 | 1.000 |
| R_transversetemporal | 179 | 0.128 | −0.019 | 0.269 | 0.088 | 1.000 |
| R_insula | 179 | −0.047 | −0.192 | 0.101 | 0.533 | 1.000 |

Table S3. Raw cortical thickness correlated with age.

| **Variable** | **N** | **r** | **CI_lower** | **CI_upper** | **p_raw** | **p_adj** |
| --- | --- | --- | --- | --- | --- | --- |
| L_precuneus_thick | 179 | −0.780 | −0.831 | −0.715 | **0.000** | **0.000** |
| R_precuneus_thick | 179 | −0.751 | −0.808 | −0.679 | **0.000** | **0.000** |
| L_superiorparietal_thick | 179 | −0.736 | −0.797 | −0.661 | **0.000** | **0.000** |
| R_inferiorparietal_thick | 179 | −0.734 | −0.795 | −0.658 | **0.000** | **0.000** |
| L_inferiorparietal_thick | 179 | −0.714 | −0.779 | −0.633 | **0.000** | **0.000** |
| L_rostralmiddlefrontal_thick | 179 | −0.712 | −0.778 | −0.632 | **0.000** | **0.000** |
| R_superiorparietal_thick | 179 | −0.712 | −0.777 | −0.631 | **0.000** | **0.000** |
| R_rostralmiddlefrontal_thick | 179 | −0.700 | −0.768 | −0.617 | **0.000** | **0.000** |
| L_superiorfrontal_thick | 179 | −0.700 | −0.767 | −0.616 | **0.000** | **0.000** |
| L_supramarginal_thick | 179 | −0.670 | −0.743 | −0.580 | **0.000** | **0.000** |
| L_lateralorbitofrontal_thick | 179 | −0.663 | −0.738 | −0.572 | **0.000** | **0.000** |
| L_lingual_thick | 179 | −0.660 | −0.735 | −0.568 | **0.000** | **0.000** |
| L_parstriangularis_thick | 179 | −0.656 | −0.732 | −0.564 | **0.000** | **0.000** |
| R_supramarginal_thick | 179 | −0.655 | −0.732 | −0.563 | **0.000** | **0.000** |
| R_superiorfrontal_thick | 179 | −0.649 | −0.727 | −0.555 | **0.000** | **0.000** |
| L_parsopercularis_thick | 179 | −0.647 | −0.725 | −0.553 | **0.000** | **0.000** |
| L_insula_thick | 179 | −0.645 | −0.723 | −0.551 | **0.000** | **0.000** |
| L_middletemporal_thick | 179 | −0.640 | −0.719 | −0.544 | **0.000** | **0.000** |
| R_lateraloccipital_thick | 179 | −0.639 | −0.719 | −0.543 | **0.000** | **0.000** |
| R_paracentral_thick | 179 | −0.632 | −0.713 | −0.535 | **0.000** | **0.000** |
| L_isthmuscingulate_thick | 179 | −0.627 | −0.709 | −0.529 | **0.000** | **0.000** |
| L_parsorbitalis_thick | 179 | −0.617 | −0.700 | −0.517 | **0.000** | **0.000** |
| L_fusiform_thick | 179 | −0.610 | −0.694 | −0.509 | **0.000** | **0.000** |
| R_parstriangularis_thick | 179 | −0.602 | −0.688 | −0.500 | **0.000** | **0.000** |
| R_bankssts_thick | 179 | −0.600 | −0.686 | −0.497 | **0.000** | **0.000** |
| R_middletemporal_thick | 179 | −0.596 | −0.683 | −0.492 | **0.000** | **0.000** |
| L_lateraloccipital_thick | 179 | −0.592 | −0.679 | −0.487 | **0.000** | **0.000** |
| R_lateralorbitofrontal_thick | 179 | −0.590 | −0.678 | −0.485 | **0.000** | **0.000** |
| L_paracentral_thick | 179 | −0.589 | −0.677 | −0.484 | **0.000** | **0.000** |
| L_transversetemporal_thick | 179 | −0.589 | −0.677 | −0.484 | **0.000** | **0.000** |
| R_insula_thick | 179 | −0.586 | −0.674 | −0.480 | **0.000** | **0.000** |
| R_posteriorcingulate_thick | 179 | −0.583 | −0.672 | −0.478 | **0.000** | **0.000** |
| L_superiortemporal_thick | 179 | −0.569 | −0.660 | −0.461 | **0.000** | **0.000** |
| R_isthmuscingulate_thick | 179 | −0.566 | −0.658 | −0.458 | **0.000** | **0.000** |
| L_postcentral_thick | 179 | −0.558 | −0.651 | −0.448 | **0.000** | **0.000** |
| L_inferiortemporal_thick | 179 | −0.557 | −0.650 | −0.446 | **0.000** | **0.000** |
| L_posteriorcingulate_thick | 179 | −0.553 | −0.647 | −0.442 | **0.000** | **0.000** |
| R_inferiortemporal_thick | 179 | −0.551 | −0.645 | −0.440 | **0.000** | **0.000** |
| L_bankssts_thick | 179 | −0.549 | −0.644 | −0.438 | **0.000** | **0.000** |
| L_rostralanteriorcingulate_thick | 179 | −0.549 | −0.644 | −0.438 | **0.000** | **0.000** |
| L_caudalmiddlefrontal_thick | 179 | −0.549 | −0.644 | −0.437 | **0.000** | **0.000** |
| R_fusiform_thick | 179 | −0.544 | −0.639 | −0.432 | **0.000** | **0.000** |
| R_lingual_thick | 179 | −0.543 | −0.639 | −0.431 | **0.000** | **0.000** |
| L_cuneus_thick | 179 | −0.533 | −0.631 | −0.420 | **0.000** | **0.000** |
| R_frontalpole_thick | 179 | −0.525 | −0.624 | −0.410 | **0.000** | **0.000** |
| R_postcentral_thick | 179 | −0.522 | −0.621 | −0.407 | **0.000** | **0.000** |
| L_frontalpole_thick | 179 | −0.520 | −0.619 | −0.404 | **0.000** | **0.000** |
| R_cuneus_thick | 179 | −0.516 | −0.616 | −0.399 | **0.000** | **0.000** |
| R_caudalmiddlefrontal_thick | 179 | −0.508 | −0.609 | −0.390 | **0.000** | **0.000** |
| L_medialorbitofrontal_thick | 179 | −0.508 | −0.609 | −0.390 | **0.000** | **0.000** |
| R_transversetemporal_thick | 179 | −0.503 | −0.605 | −0.385 | **0.000** | **0.000** |
| R_parsopercularis_thick | 179 | −0.491 | −0.594 | −0.371 | **0.000** | **0.000** |
| R_superiortemporal_thick | 179 | −0.487 | −0.591 | −0.366 | **0.000** | **0.000** |
| R_medialorbitofrontal_thick | 179 | −0.479 | −0.584 | −0.357 | **0.000** | **0.000** |
| R_parsorbitalis_thick | 179 | −0.461 | −0.569 | −0.338 | **0.000** | **0.000** |
| L_caudalanteriorcingulate_thick | 179 | −0.461 | −0.569 | −0.337 | **0.000** | **0.000** |
| R_caudalanteriorcingulate_thick | 179 | −0.455 | −0.564 | −0.330 | **0.000** | **0.000** |
| L_precentral_thick | 179 | −0.408 | −0.524 | −0.278 | **0.000** | **0.000** |
| R_rostralanteriorcingulate_thick | 179 | −0.405 | −0.521 | −0.275 | **0.000** | **0.000** |
| L_pericalcarine_thick | 179 | −0.322 | −0.448 | −0.184 | **0.000** | **0.001** |
| R_precentral_thick | 179 | −0.288 | −0.417 | −0.147 | **0.000** | **0.007** |
| R_parahippocampal_thick | 179 | −0.283 | −0.412 | −0.142 | **0.000** | **0.009** |
| L_entorhinal_thick | 179 | 0.247 | 0.104 | 0.380 | **0.001** | 0.059 |
| R_pericalcarine_thick | 179 | −0.209 | −0.345 | −0.064 | **0.005** | 0.350 |
| L_parahippocampal_thick | 179 | −0.175 | −0.314 | −0.029 | **0.019** | 1.000 |
| L_temporalpole_thick | 179 | 0.014 | −0.133 | 0.160 | 0.856 | 1.000 |
| R_entorhinal_thick | 179 | 0.070 | −0.078 | 0.214 | 0.354 | 1.000 |
| R_temporalpole_thick | 179 | 0.134 | −0.013 | 0.275 | 0.074 | 1.000 |

Table S4. A) Raw cortical volume difference between genders.

| **Variable** | **estimate** | **std.error** | **statistic** | **p.value** | **conf.low** | **conf.high** | **p_adj** | **Cohen_d** |
| --- | --- | --- | --- | --- | --- | --- | --- | --- |
| lhCortexVol | 25781.512 | 3425.40 | 7.53 | **0.00** | 19019.71 | 32543.31 | **0.000** | 1.143 |
| rhCortexVol | 25634.021 | 3392.45 | 7.56 | **0.00** | 18937.27 | 32330.78 | **0.000** | 1.148 |
| CortexVol | 51415.533 | 6797.61 | 7.56 | **0.00** | 37996.94 | 64834.13 | **0.000** | 1.149 |
| lhCerebralWhiteMatterVol | 23154.333 | 3335.00 | 6.94 | **0.00** | 16570.99 | 29737.68 | **0.000** | 1.055 |
| rhCerebralWhiteMatterVol | 23215.510 | 3280.89 | 7.08 | **0.00** | 16738.97 | 29692.05 | **0.000** | 1.075 |
| CerebralWhiteMatterVol | 46369.843 | 6604.25 | 7.02 | **0.00** | 33332.95 | 59406.74 | **0.000** | 1.067 |
| Left_Cerebellum_Cortex | 4925.213 | 687.04 | 7.17 | **0.00** | 3568.99 | 6281.43 | **0.000** | 1.089 |
| Right_Cerebellum_Cortex | 4560.538 | 710.94 | 6.41 | **0.00** | 3157.14 | 5963.94 | **0.000** | 0.975 |
| Left_Cerebellum_WM | 774.578 | 196.18 | 3.95 | **0.00** | 387.31 | 1161.84 | **0.005** | 0.600 |
| Right_Cerebellum_WM | 740.753 | 183.87 | 4.03 | **0.00** | 377.79 | 1103.71 | **0.004** | 0.612 |
| SubCortGrayVol | 4491.292 | 671.19 | 6.69 | **0.00** | 3166.35 | 5816.24 | **0.000** | 1.017 |
| Left_Amygdala | 155.153 | 28.77 | 5.39 | **0.00** | 98.37 | 211.94 | **0.000** | 0.819 |
| Right_Amygdala | 177.904 | 29.17 | 6.10 | **0.00** | 120.32 | 235.49 | **0.000** | 0.926 |
| Left_Thalamus | 623.028 | 112.67 | 5.53 | **0.00** | 400.62 | 845.44 | **0.000** | 0.840 |
| Right_Thalamus | 696.437 | 104.59 | 6.66 | **0.00** | 489.97 | 902.90 | **0.000** | 1.012 |
| Left_Putamen | 435.044 | 87.77 | 4.96 | **0.00** | 261.77 | 608.31 | **0.000** | 0.753 |
| Right_Putamen | 447.133 | 85.33 | 5.24 | **0.00** | 278.70 | 615.57 | **0.000** | 0.796 |
| Left_Pallidum | 155.590 | 29.13 | 5.34 | **0.00** | 98.09 | 213.10 | **0.000** | 0.811 |
| Right_Pallidum | 163.645 | 30.63 | 5.34 | **0.00** | 103.19 | 224.11 | **0.000** | 0.812 |
| Left_Hippocampus | 174.021 | 51.62 | 3.37 | **0.00** | 72.13 | 275.91 | **0.043** | 0.512 |
| Right_Hippocampus | 266.441 | 54.03 | 4.93 | **0.00** | 159.79 | 373.09 | **0.000** | 0.749 |
| Left_Accumbens_area | 4.783 | 15.98 | 0.30 | 0.77 | -26.77 | 36.33 | 1.000 | 0.045 |
| Right_Accumbens_area | 31.854 | 14.32 | 2.23 | **0.03** | 3.59 | 60.11 | 1.000 | 0.338 |
| Left_Caudate | 229.009 | 78.45 | 2.92 | **0.00** | 74.14 | 383.88 | 0.183 | 0.443 |
| Right_Caudate | 250.448 | 79.28 | 3.16 | **0.00** | 93.94 | 406.96 | 0.086 | 0.480 |
| Left_VentralDC | 270.104 | 49.76 | 5.43 | 0.00 | 171.88 | 368.32 | **0.000** | 0.825 |
| Right_VentralDC | 310.207 | 50.75 | 6.11 | 0.00 | 210.03 | 410.38 | **0.000** | 0.929 |
| Brain_Stem | 1649.438 | 251.88 | 6.55 | 0.00 | 1152.22 | 2146.65 | **0.000** | 0.995 |

Table S4. B) Scaled cortical volume difference between genders.

| **Variable** | **estimate** | **std.error** | **statistic** | **p.value** | **conf.low** | **conf.high** | **p_adj** | **Cohen_d** |
| --- | --- | --- | --- | --- | --- | --- | --- | --- |
| lhCortexVol | 0.017 | 0.002 | 7.456 | 0.000 | 0.012 | 0.022 | **0.000** | 1.133 |
| rhCortexVol | 0.017 | 0.002 | 7.481 | 0.000 | 0.012 | 0.021 | **0.000** | 1.136 |
| CortexVol | 0.034 | 0.005 | 7.490 | 0.000 | 0.025 | 0.043 | **0.000** | 1.138 |
| lhCerebralWhiteMatterVol | 0.015 | 0.002 | 6.683 | 0.000 | 0.010 | 0.019 | **0.000** | 1.015 |
| rhCerebralWhiteMatterVol | 0.015 | 0.002 | 6.813 | 0.000 | 0.011 | 0.019 | **0.000** | 1.035 |
| CerebralWhiteMatterVol | 0.030 | 0.004 | 6.759 | 0.000 | 0.021 | 0.038 | **0.000** | 1.027 |
| Left_Cerebellum_Cortex | 0.003 | 0.000 | 7.071 | 0.000 | 0.002 | 0.004 | **0.000** | 1.074 |
| Right_Cerebellum_Cortex | 0.003 | 0.000 | 6.260 | 0.000 | 0.002 | 0.004 | **0.000** | 0.951 |
| Left_Cerebellum_WM | 0.000 | 0.000 | 3.688 | 0.000 | 0.000 | 0.001 | **0.014** | 0.560 |
| Right_Cerebellum_WM | 0.000 | 0.000 | 3.804 | 0.000 | 0.000 | 0.001 | **0.009** | 0.578 |
| SubCortGrayVol | 0.003 | 0.000 | 6.257 | 0.000 | 0.002 | 0.004 | **0.000** | 0.951 |
| Left_Amygdala | 0.000 | 0.000 | 5.289 | 0.000 | 0.000 | 0.000 | **0.000** | 0.803 |
| Right_Amygdala | 0.000 | 0.000 | 5.935 | 0.000 | 0.000 | 0.000 | **0.000** | 0.902 |
| Left_Thalamus | 0.000 | 0.000 | 5.334 | 0.000 | 0.000 | 0.001 | **0.000** | 0.810 |
| Right_Thalamus | 0.000 | 0.000 | 6.485 | 0.000 | 0.000 | 0.001 | **0.000** | 0.985 |
| Left_Putamen | 0.000 | 0.000 | 4.699 | 0.000 | 0.000 | 0.000 | **0.000** | 0.714 |
| Right_Putamen | 0.000 | 0.000 | 4.966 | 0.000 | 0.000 | 0.000 | **0.000** | 0.754 |
| Left_Pallidum | 0.000 | 0.000 | 5.092 | 0.000 | 0.000 | 0.000 | **0.000** | 0.774 |
| Right_Pallidum | 0.000 | 0.000 | 5.112 | 0.000 | 0.000 | 0.000 | **0.000** | 0.777 |
| Left_Hippocampus | 0.000 | 0.000 | 3.115 | 0.002 | 0.000 | 0.000 | **0.102** | 0.473 |
| Right_Hippocampus | 0.000 | 0.000 | 4.680 | 0.000 | 0.000 | 0.000 | **0.000** | 0.711 |
| Left_Accumbens_area | 0.000 | 0.000 | 0.155 | 0.877 | 0.000 | 0.000 | 1.000 | 0.024 |
| Right_Accumbens_area | 0.000 | 0.000 | 2.065 | 0.040 | 0.000 | 0.000 | 1.000 | 0.314 |
| Left_Caudate | 0.000 | 0.000 | 2.720 | 0.007 | 0.000 | 0.000 | 0.339 | 0.413 |
| Right_Caudate | 0.000 | 0.000 | 2.939 | 0.004 | 0.000 | 0.000 | 0.177 | 0.446 |
| Left_VentralDC | 0.000 | 0.000 | 5.126 | 0.000 | 0.000 | 0.000 | **0.000** | 0.779 |
| Right_VentralDC | 0.000 | 0.000 | 5.805 | 0.000 | 0.000 | 0.000 | **0.000** | 0.882 |
| Brain_Stem | 0.001 | 0.000 | 6.246 | 0.000 | 0.001 | 0.001 | **0.000** | 0.949 |

Table S5. A) Cortical thickness difference between genders adjusted for age, but without ICV correction.

| **Variable** | **estimate** | **std.error** | **statistic** | **p.value** | **conf.low** | **conf.high** | **p_adj** | **Cohen_d** |
| --- | --- | --- | --- | --- | --- | --- | --- | --- |
| L_bankssts_thick | -0.016 | 0.020 | -0.770 | 0.442 | -0.056 | 0.024 | 1.000 | -0.115 |
| L_caudalanteriorcingulate_thick | -0.008 | 0.028 | -0.268 | 0.789 | -0.063 | 0.048 | 1.000 | -0.040 |
| L_caudalmiddlefrontal_thick | 0.013 | 0.016 | 0.798 | 0.426 | -0.019 | 0.045 | 1.000 | 0.119 |
| L_cuneus_thick | 0.012 | 0.022 | 0.551 | 0.582 | -0.031 | 0.055 | 1.000 | 0.083 |
| L_entorhinal_thick | -0.022 | 0.038 | -0.564 | 0.573 | -0.098 | 0.054 | 1.000 | -0.084 |
| L_fusiform_thick | 0.000 | 0.015 | 0.013 | 0.990 | -0.029 | 0.029 | 1.000 | 0.002 |
| L_inferiorparietal_thick | -0.001 | 0.016 | -0.052 | 0.959 | -0.033 | 0.031 | 1.000 | -0.008 |
| L_inferiortemporal_thick | 0.003 | 0.019 | 0.170 | 0.865 | -0.035 | 0.041 | 1.000 | 0.025 |
| L_isthmuscingulate_thick | 0.009 | 0.020 | 0.456 | 0.649 | -0.030 | 0.048 | 1.000 | 0.068 |
| L_lateraloccipital_thick | 0.035 | 0.016 | 2.199 | 0.029 | 0.004 | 0.066 | 1.000 | 0.329 |
| L_lateralorbitofrontal_thick | -0.004 | 0.016 | -0.232 | 0.816 | -0.036 | 0.028 | 1.000 | -0.035 |
| L_lingual_thick | -0.018 | 0.018 | -1.015 | 0.312 | -0.054 | 0.017 | 1.000 | -0.152 |
| L_medialorbitofrontal_thick | 0.026 | 0.018 | 1.402 | 0.163 | -0.011 | 0.062 | 1.000 | 0.210 |
| L_middletemporal_thick | -0.011 | 0.017 | -0.614 | 0.540 | -0.044 | 0.023 | 1.000 | -0.092 |
| L_parahippocampal_thick | -0.093 | 0.036 | -2.609 | **0.010** | -0.164 | -0.023 | 0.671 | -0.391 |
| L_paracentral_thick | -0.023 | 0.019 | -1.242 | 0.216 | -0.060 | 0.014 | 1.000 | -0.186 |
| L_parsopercularis_thick | 0.017 | 0.015 | 1.118 | 0.265 | -0.013 | 0.047 | 1.000 | 0.167 |
| L_parsorbitalis_thick | -0.044 | 0.025 | -1.808 | 0.072 | -0.093 | 0.004 | 1.000 | -0.271 |
| L_parstriangularis_thick | 0.008 | 0.017 | 0.436 | 0.664 | -0.027 | 0.042 | 1.000 | 0.065 |
| L_pericalcarine_thick | -0.001 | 0.025 | -0.029 | 0.977 | -0.050 | 0.049 | 1.000 | -0.004 |
| L_postcentral_thick | -0.039 | 0.018 | -2.205 | 0.029 | -0.074 | -0.004 | 1.000 | -0.330 |
| L_posteriorcingulate_thick | 0.024 | 0.019 | 1.230 | 0.220 | -0.014 | 0.062 | 1.000 | 0.184 |
| L_precentral_thick | -0.027 | 0.015 | -1.738 | 0.084 | -0.057 | 0.004 | 1.000 | -0.260 |
| L_precuneus_thick | 0.009 | 0.014 | 0.640 | 0.523 | -0.018 | 0.036 | 1.000 | 0.096 |
| L_rostralanteriorcingulate_thick | 0.006 | 0.028 | 0.224 | 0.823 | -0.049 | 0.061 | 1.000 | 0.033 |
| L_rostralmiddlefrontal_thick | 0.034 | 0.014 | 2.466 | **0.015** | 0.007 | 0.061 | 0.995 | 0.369 |
| L_superiorfrontal_thick | 0.004 | 0.016 | 0.252 | 0.802 | -0.027 | 0.035 | 1.000 | 0.038 |
| L_superiorparietal_thick | 0.007 | 0.014 | 0.470 | 0.639 | -0.021 | 0.034 | 1.000 | 0.070 |
| L_superiortemporal_thick | -0.020 | 0.018 | -1.074 | 0.284 | -0.056 | 0.017 | 1.000 | -0.161 |
| L_supramarginal_thick | -0.030 | 0.016 | -1.886 | 0.061 | -0.062 | 0.001 | 1.000 | -0.282 |
| L_frontalpole_thick | 0.032 | 0.034 | 0.946 | 0.345 | -0.035 | 0.098 | 1.000 | 0.142 |
| L_temporalpole_thick | -0.075 | 0.042 | -1.776 | 0.077 | -0.158 | 0.008 | 1.000 | -0.266 |
| L_transversetemporal_thick | -0.033 | 0.023 | -1.421 | 0.157 | -0.079 | 0.013 | 1.000 | -0.213 |
| L_insula_thick | 0.013 | 0.017 | 0.801 | 0.424 | -0.020 | 0.046 | 1.000 | 0.120 |
| R_bankssts_thick | -0.004 | 0.022 | -0.183 | 0.855 | -0.048 | 0.040 | 1.000 | -0.027 |
| R_caudalanteriorcingulate_thick | -0.007 | 0.027 | -0.254 | 0.800 | -0.059 | 0.046 | 1.000 | -0.038 |
| R_caudalmiddlefrontal_thick | 0.006 | 0.017 | 0.336 | 0.737 | -0.028 | 0.040 | 1.000 | 0.050 |
| R_cuneus_thick | -0.004 | 0.021 | -0.178 | 0.859 | -0.046 | 0.038 | 1.000 | -0.027 |
| R_entorhinal_thick | -0.051 | 0.047 | -1.101 | 0.272 | -0.144 | 0.041 | 1.000 | -0.165 |
| R_fusiform_thick | 0.002 | 0.017 | 0.117 | 0.907 | -0.032 | 0.036 | 1.000 | 0.018 |
| R_inferiorparietal_thick | 0.001 | 0.015 | 0.075 | 0.940 | -0.029 | 0.032 | 1.000 | 0.011 |
| R_inferiortemporal_thick | -0.001 | 0.018 | -0.055 | 0.956 | -0.037 | 0.035 | 1.000 | -0.008 |
| R_isthmuscingulate_thick | 0.003 | 0.022 | 0.129 | 0.898 | -0.041 | 0.046 | 1.000 | 0.019 |
| R_lateraloccipital_thick | 0.018 | 0.016 | 1.124 | 0.262 | -0.013 | 0.049 | 1.000 | 0.168 |
| R_lateralorbitofrontal_thick | 0.011 | 0.018 | 0.606 | 0.545 | -0.024 | 0.046 | 1.000 | 0.091 |
| R_lingual_thick | 0.011 | 0.023 | 0.464 | 0.643 | -0.034 | 0.055 | 1.000 | 0.070 |
| R_medialorbitofrontal_thick | 0.020 | 0.018 | 1.088 | 0.278 | -0.016 | 0.056 | 1.000 | 0.163 |
| R_middletemporal_thick | 0.002 | 0.018 | 0.089 | 0.930 | -0.033 | 0.036 | 1.000 | 0.013 |
| R_parahippocampal_thick | -0.064 | 0.027 | -2.337 | 0.021 | -0.118 | -0.010 | 1.000 | -0.350 |
| R_paracentral_thick | -0.001 | 0.017 | -0.034 | 0.973 | -0.035 | 0.033 | 1.000 | -0.005 |
| R_parsopercularis_thick | 0.020 | 0.019 | 1.087 | 0.278 | -0.017 | 0.057 | 1.000 | 0.163 |
| R_parsorbitalis_thick | -0.060 | 0.023 | -2.621 | **0.010** | -0.105 | -0.015 | 0.648 | -0.392 |
| R_parstriangularis_thick | -0.008 | 0.017 | -0.443 | 0.658 | -0.041 | 0.026 | 1.000 | -0.066 |
| R_pericalcarine_thick | -0.010 | 0.023 | -0.454 | 0.650 | -0.056 | 0.035 | 1.000 | -0.068 |
| R_postcentral_thick | -0.036 | 0.015 | -2.310 | **0.022** | -0.066 | -0.005 | 1.000 | -0.346 |
| R_posteriorcingulate_thick | 0.028 | 0.016 | 1.673 | 0.096 | -0.005 | 0.060 | 1.000 | 0.250 |
| R_precentral_thick | -0.024 | 0.017 | -1.347 | 0.180 | -0.058 | 0.011 | 1.000 | -0.202 |
| R_precuneus_thick | 0.020 | 0.015 | 1.365 | 0.174 | -0.009 | 0.050 | 1.000 | 0.204 |
| R_rostralanteriorcingulate_thick | -0.050 | 0.032 | -1.563 | 0.120 | -0.114 | 0.013 | 1.000 | -0.234 |
| R_rostralmiddlefrontal_thick | 0.019 | 0.014 | 1.347 | 0.180 | -0.009 | 0.048 | 1.000 | 0.202 |
| R_superiorfrontal_thick | 0.001 | 0.017 | 0.034 | 0.973 | -0.033 | 0.034 | 1.000 | 0.005 |
| R_superiorparietal_thick | -0.001 | 0.014 | -0.060 | 0.953 | -0.029 | 0.028 | 1.000 | -0.009 |
| R_superiortemporal_thick | -0.030 | 0.021 | -1.440 | 0.152 | -0.071 | 0.011 | 1.000 | -0.216 |
| R_supramarginal_thick | -0.016 | 0.015 | -1.030 | 0.305 | -0.046 | 0.014 | 1.000 | -0.154 |
| R_frontalpole_thick | 0.023 | 0.034 | 0.676 | 0.500 | -0.044 | 0.090 | 1.000 | 0.101 |
| R_temporalpole_thick | -0.022 | 0.042 | -0.535 | 0.594 | -0.104 | 0.060 | 1.000 | -0.080 |
| R_transversetemporal_thick | 0.002 | 0.027 | 0.061 | 0.952 | -0.051 | 0.055 | 1.000 | 0.009 |
| R_insula_thick | 0.032 | 0.017 | 1.861 | 0.064 | -0.002 | 0.066 | 1.000 | 0.279 |

Table S5. B) Cortical thickness difference between genders adjusted for age and ICV.

| **Variable** | **estimate** | **std.error** | **statistic** | **p.value** | **conf.low** | **conf.high** | **p_adj** | **Cohen_d** |
| --- | --- | --- | --- | --- | --- | --- | --- | --- |
| L_bankssts_thick | -0.014 | 0.020 | -0.663 | 0.508 | -0.054 | 0.027 | 1.000 | -0.099 |
| L_caudalanteriorcingulate_thick | -0.010 | 0.028 | -0.360 | 0.719 | -0.066 | 0.046 | 1.000 | -0.054 |
| L_caudalmiddlefrontal_thick | 0.013 | 0.016 | 0.785 | 0.434 | -0.019 | 0.045 | 1.000 | 0.117 |
| L_cuneus_thick | 0.013 | 0.022 | 0.586 | 0.559 | -0.030 | 0.055 | 1.000 | 0.088 |
| L_entorhinal_thick | -0.021 | 0.038 | -0.539 | 0.591 | -0.096 | 0.055 | 1.000 | -0.081 |
| L_fusiform_thick | 0.001 | 0.015 | 0.036 | 0.971 | -0.028 | 0.029 | 1.000 | 0.005 |
| L_inferiorparietal_thick | -0.001 | 0.016 | -0.076 | 0.939 | -0.033 | 0.031 | 1.000 | -0.011 |
| L_inferiortemporal_thick | 0.003 | 0.019 | 0.178 | 0.859 | -0.034 | 0.041 | 1.000 | 0.027 |
| L_isthmuscingulate_thick | 0.010 | 0.020 | 0.510 | 0.611 | -0.029 | 0.049 | 1.000 | 0.076 |
| L_lateraloccipital_thick | 0.036 | 0.016 | 2.275 | 0.024 | 0.005 | 0.068 | 1.000 | 0.340 |
| L_lateralorbitofrontal_thick | -0.005 | 0.016 | -0.302 | 0.763 | -0.037 | 0.027 | 1.000 | -0.045 |
| L_lingual_thick | -0.017 | 0.018 | -0.947 | 0.345 | -0.053 | 0.019 | 1.000 | -0.142 |
| L_medialorbitofrontal_thick | 0.029 | 0.019 | 1.514 | 0.132 | -0.009 | 0.066 | 1.000 | 0.226 |
| L_middletemporal_thick | -0.010 | 0.017 | -0.604 | 0.547 | -0.044 | 0.023 | 1.000 | -0.090 |
| L_parahippocampal_thick | -0.094 | 0.036 | -2.634 | 0.009 | -0.164 | -0.024 | 0.626 | -0.394 |
| L_paracentral_thick | -0.023 | 0.019 | -1.253 | 0.212 | -0.060 | 0.013 | 1.000 | -0.187 |
| L_parsopercularis_thick | 0.017 | 0.015 | 1.103 | 0.271 | -0.013 | 0.046 | 1.000 | 0.165 |
| L_parsorbitalis_thick | -0.046 | 0.025 | -1.859 | 0.065 | -0.094 | 0.003 | 1.000 | -0.278 |
| L_parstriangularis_thick | 0.009 | 0.018 | 0.528 | 0.598 | -0.025 | 0.044 | 1.000 | 0.079 |
| L_pericalcarine_thick | -0.002 | 0.025 | -0.066 | 0.947 | -0.051 | 0.048 | 1.000 | -0.010 |
| L_postcentral_thick | -0.037 | 0.018 | -2.073 | 0.040 | -0.072 | -0.002 | 1.000 | -0.310 |
| L_posteriorcingulate_thick | 0.025 | 0.019 | 1.278 | 0.203 | -0.013 | 0.062 | 1.000 | 0.191 |
| L_precentral_thick | -0.027 | 0.015 | -1.748 | 0.082 | -0.057 | 0.003 | 1.000 | -0.261 |
| L_precuneus_thick | 0.011 | 0.014 | 0.781 | 0.436 | -0.017 | 0.039 | 1.000 | 0.117 |
| L_rostralanteriorcingulate_thick | 0.007 | 0.028 | 0.251 | 0.802 | -0.048 | 0.062 | 1.000 | 0.038 |
| L_rostralmiddlefrontal_thick | 0.036 | 0.014 | 2.553 | 0.012 | 0.008 | 0.063 | 0.783 | 0.382 |
| L_superiorfrontal_thick | 0.004 | 0.016 | 0.274 | 0.785 | -0.027 | 0.036 | 1.000 | 0.041 |
| L_superiorparietal_thick | 0.008 | 0.014 | 0.568 | 0.571 | -0.020 | 0.036 | 1.000 | 0.085 |
| L_superiortemporal_thick | -0.020 | 0.018 | -1.083 | 0.280 | -0.056 | 0.016 | 1.000 | -0.162 |
| L_supramarginal_thick | -0.030 | 0.016 | -1.884 | 0.061 | -0.061 | 0.001 | 1.000 | -0.282 |
| L_frontalpole_thick | 0.033 | 0.033 | 0.984 | 0.326 | -0.033 | 0.099 | 1.000 | 0.147 |
| L_temporalpole_thick | -0.079 | 0.042 | -1.863 | 0.064 | -0.162 | 0.005 | 1.000 | -0.278 |
| L_transversetemporal_thick | -0.032 | 0.023 | -1.367 | 0.173 | -0.077 | 0.014 | 1.000 | -0.204 |
| L_insula_thick | 0.013 | 0.017 | 0.789 | 0.431 | -0.020 | 0.046 | 1.000 | 0.118 |
| R_bankssts_thick | -0.001 | 0.023 | -0.045 | 0.964 | -0.046 | 0.043 | 1.000 | -0.007 |
| R_caudalanteriorcingulate_thick | -0.007 | 0.027 | -0.281 | 0.779 | -0.060 | 0.045 | 1.000 | -0.042 |
| R_caudalmiddlefrontal_thick | 0.008 | 0.018 | 0.460 | 0.646 | -0.027 | 0.043 | 1.000 | 0.069 |
| R_cuneus_thick | -0.003 | 0.021 | -0.141 | 0.888 | -0.045 | 0.039 | 1.000 | -0.021 |
| R_entorhinal_thick | -0.051 | 0.046 | -1.095 | 0.275 | -0.142 | 0.041 | 1.000 | -0.164 |
| R_fusiform_thick | 0.002 | 0.017 | 0.145 | 0.885 | -0.031 | 0.036 | 1.000 | 0.022 |
| R_inferiorparietal_thick | 0.004 | 0.016 | 0.246 | 0.806 | -0.028 | 0.035 | 1.000 | 0.037 |
| R_inferiortemporal_thick | 0.000 | 0.019 | 0.005 | 0.996 | -0.036 | 0.037 | 1.000 | 0.001 |
| R_isthmuscingulate_thick | 0.005 | 0.022 | 0.220 | 0.826 | -0.039 | 0.049 | 1.000 | 0.033 |
| R_lateraloccipital_thick | 0.019 | 0.016 | 1.206 | 0.230 | -0.012 | 0.050 | 1.000 | 0.180 |
| R_lateralorbitofrontal_thick | 0.010 | 0.018 | 0.549 | 0.584 | -0.025 | 0.045 | 1.000 | 0.082 |
| R_lingual_thick | 0.012 | 0.023 | 0.536 | 0.593 | -0.032 | 0.057 | 1.000 | 0.080 |
| R_medialorbitofrontal_thick | 0.021 | 0.018 | 1.169 | 0.244 | -0.015 | 0.057 | 1.000 | 0.175 |
| R_middletemporal_thick | 0.002 | 0.018 | 0.126 | 0.900 | -0.033 | 0.037 | 1.000 | 0.019 |
| R_parahippocampal_thick | -0.065 | 0.027 | -2.362 | 0.019 | -0.118 | -0.011 | 1.000 | -0.353 |
| R_paracentral_thick | 0.001 | 0.017 | 0.047 | 0.962 | -0.033 | 0.035 | 1.000 | 0.007 |
| R_parsopercularis_thick | 0.021 | 0.019 | 1.108 | 0.269 | -0.016 | 0.058 | 1.000 | 0.166 |
| R_parsorbitalis_thick | -0.060 | 0.023 | -2.632 | 0.009 | -0.105 | -0.015 | 0.628 | -0.394 |
| R_parstriangularis_thick | -0.006 | 0.017 | -0.331 | 0.741 | -0.040 | 0.028 | 1.000 | -0.049 |
| R_pericalcarine_thick | -0.012 | 0.023 | -0.530 | 0.597 | -0.058 | 0.033 | 1.000 | -0.079 |
| R_postcentral_thick | -0.033 | 0.016 | -2.057 | 0.041 | -0.064 | -0.001 | 1.000 | -0.308 |
| R_posteriorcingulate_thick | 0.028 | 0.016 | 1.702 | 0.091 | -0.004 | 0.060 | 1.000 | 0.254 |
| R_precentral_thick | -0.024 | 0.017 | -1.379 | 0.170 | -0.058 | 0.010 | 1.000 | -0.206 |
| R_precuneus_thick | 0.023 | 0.015 | 1.483 | 0.140 | -0.008 | 0.053 | 1.000 | 0.222 |
| R_rostralanteriorcingulate_thick | -0.052 | 0.032 | -1.611 | 0.109 | -0.115 | 0.012 | 1.000 | -0.241 |
| R_rostralmiddlefrontal_thick | 0.020 | 0.014 | 1.423 | 0.157 | -0.008 | 0.049 | 1.000 | 0.213 |
| R_superiorfrontal_thick | 0.001 | 0.017 | 0.051 | 0.959 | -0.033 | 0.035 | 1.000 | 0.008 |
| R_superiorparietal_thick | 0.001 | 0.015 | 0.086 | 0.931 | -0.028 | 0.030 | 1.000 | 0.013 |
| R_superiortemporal_thick | -0.030 | 0.021 | -1.431 | 0.154 | -0.071 | 0.011 | 1.000 | -0.214 |
| R_supramarginal_thick | -0.015 | 0.015 | -0.961 | 0.338 | -0.045 | 0.016 | 1.000 | -0.144 |
| R_frontalpole_thick | 0.024 | 0.034 | 0.722 | 0.471 | -0.042 | 0.091 | 1.000 | 0.108 |
| R_temporalpole_thick | -0.020 | 0.042 | -0.488 | 0.626 | -0.102 | 0.062 | 1.000 | -0.073 |
| R_transversetemporal_thick | 0.002 | 0.027 | 0.091 | 0.928 | -0.050 | 0.055 | 1.000 | 0.014 |
| R_insula_thick | 0.031 | 0.017 | 1.790 | 0.075 | -0.003 | 0.065 | 1.000 | 0.268 |

Table S6. A) Cortical area difference between genders adjusted for age only.

| **ROI name** | **estimate** | **std.error** | **statistic** | **p.value** | **conf.low** | **conf.high** | **p_adj** | **Cohen_d** |
| --- | --- | --- | --- | --- | --- | --- | --- | --- |
| L_bankssts | 95.780 | 27.283 | 3.511 | 0.001 | 41.937 | 149.624 | **0.039** | 0.525 |
| L_caudalanteriorcingulate | 40.933 | 20.238 | 2.023 | 0.045 | 0.993 | 80.873 | 1.000 | 0.302 |
| L_caudalmiddlefrontal | 285.212 | 55.810 | 5.110 | 0.000 | 175.070 | 395.354 | **0.000** | 0.764 |
| L_cuneus | 156.737 | 35.190 | 4.454 | 0.000 | 87.288 | 226.186 | **0.001** | 0.666 |
| L_entorhinal | 43.126 | 15.457 | 2.790 | 0.006 | 12.621 | 73.630 | 0.398 | 0.417 |
| L_fusiform | 366.449 | 56.051 | 6.538 | 0.000 | 255.829 | 477.068 | **0.000** | 0.978 |
| L_inferiorparietal | 419.069 | 102.546 | 4.087 | 0.000 | 216.692 | 621.447 | **0.005** | 0.611 |
| L_inferiortemporal | 407.599 | 66.239 | 6.153 | 0.000 | 276.874 | 538.324 | **0.000** | 0.920 |
| L_isthmuscingulate | 92.756 | 23.822 | 3.894 | 0.000 | 45.742 | 139.769 | **0.010** | 0.582 |
| L_lateraloccipital | 557.407 | 89.780 | 6.209 | 0.000 | 380.224 | 734.590 | **0.000** | 0.928 |
| L_lateralorbitofrontal | 246.960 | 42.630 | 5.793 | 0.000 | 162.829 | 331.092 | **0.000** | 0.866 |
| L_lingual | 317.374 | 57.352 | 5.534 | 0.000 | 204.188 | 430.560 | **0.000** | 0.827 |
| L_medialorbitofrontal | 169.351 | 36.170 | 4.682 | 0.000 | 97.968 | 240.734 | **0.000** | 0.700 |
| L_middletemporal | 347.276 | 64.254 | 5.405 | 0.000 | 220.470 | 474.083 | **0.000** | 0.808 |
| L_parahippocampal | 46.098 | 12.555 | 3.672 | 0.000 | 21.321 | 70.876 | **0.022** | 0.549 |
| L_paracentral | 112.323 | 25.080 | 4.479 | 0.000 | 62.827 | 161.818 | **0.001** | 0.670 |
| L_parsopercularis | 199.270 | 41.550 | 4.796 | 0.000 | 117.269 | 281.272 | **0.000** | 0.717 |
| L_parsorbitalis | 102.129 | 14.515 | 7.036 | 0.000 | 73.482 | 130.776 | **0.000** | 1.052 |
| L_parstriangularis | 165.982 | 37.656 | 4.408 | 0.000 | 91.667 | 240.297 | **0.001** | 0.659 |
| L_pericalcarine | 137.550 | 37.746 | 3.644 | 0.000 | 63.056 | 212.043 | **0.024** | 0.545 |
| L_postcentral | 517.616 | 79.139 | 6.541 | 0.000 | 361.433 | 673.799 | **0.000** | 0.978 |
| L_posteriorcingulate | 135.045 | 26.168 | 5.161 | 0.000 | 83.401 | 186.690 | **0.000** | 0.772 |
| L_precentral | 542.349 | 72.977 | 7.432 | 0.000 | 398.326 | 686.372 | **0.000** | 1.111 |
| L_precuneus | 462.962 | 75.349 | 6.144 | 0.000 | 314.259 | 611.665 | **0.000** | 0.919 |
| L_rostralanteriorcingulate | 78.632 | 24.827 | 3.167 | 0.002 | 29.634 | 127.629 | 0.123 | 0.474 |
| L_rostralmiddlefrontal | 794.584 | 110.016 | 7.222 | 0.000 | 577.463 | 1011.704 | **0.000** | 1.080 |
| L_superiorfrontal | 923.912 | 132.887 | 6.953 | 0.000 | 661.654 | 1186.169 | **0.000** | 1.040 |
| L_superiorparietal | 514.884 | 115.664 | 4.452 | 0.000 | 286.618 | 743.151 | **0.001** | 0.666 |
| L_superiortemporal | 495.892 | 71.130 | 6.972 | 0.000 | 355.514 | 636.269 | **0.000** | 1.042 |
| L_supramarginal | 576.364 | 109.776 | 5.250 | 0.000 | 359.717 | 793.012 | **0.000** | 0.785 |
| L_frontalpole | 30.096 | 5.625 | 5.351 | 0.000 | 18.996 | 41.196 | **0.000** | 0.800 |
| L_temporalpole | 32.621 | 10.204 | 3.197 | 0.002 | 12.482 | 52.760 | **0.112** | 0.478 |
| L_transversetemporal | 50.643 | 9.984 | 5.073 | 0.000 | 30.940 | 70.346 | **0.000** | 0.758 |
| L_insula | 160.266 | 38.845 | 4.126 | 0.000 | 83.604 | 236.928 | **0.004** | 0.617 |
| R_bankssts | 100.524 | 19.332 | 5.200 | 0.000 | 62.370 | 138.677 | **0.000** | 0.777 |
| R_caudalanteriorcingulate | 53.990 | 20.107 | 2.685 | 0.008 | 14.309 | 93.672 | 0.540 | 0.401 |
| R_caudalmiddlefrontal | 187.329 | 53.148 | 3.525 | 0.001 | 82.440 | 292.218 | **0.037** | 0.527 |
| R_cuneus | 165.203 | 33.259 | 4.967 | 0.000 | 99.566 | 230.840 | **0.000** | 0.743 |
| R_entorhinal | 55.877 | 13.109 | 4.263 | 0.000 | 30.006 | 81.748 | **0.002** | 0.637 |
| R_fusiform | 383.171 | 52.779 | 7.260 | 0.000 | 279.010 | 487.332 | **0.000** | 1.085 |
| R_inferiorparietal | 650.550 | 123.726 | 5.258 | 0.000 | 406.373 | 894.727 | **0.000** | 0.786 |
| R_inferiortemporal | 394.231 | 61.158 | 6.446 | 0.000 | 273.535 | 514.928 | **0.000** | 0.964 |
| R_isthmuscingulate | 92.990 | 22.494 | 4.134 | 0.000 | 48.596 | 137.383 | **0.004** | 0.618 |
| R_lateraloccipital | 579.498 | 83.681 | 6.925 | 0.000 | 414.350 | 744.645 | **0.000** | 1.035 |
| R_lateralorbitofrontal | 240.022 | 52.044 | 4.612 | 0.000 | 137.312 | 342.732 | **0.001** | 0.690 |
| R_lingual | 274.961 | 64.136 | 4.287 | 0.000 | 148.387 | 401.535 | **0.002** | 0.641 |
| R_medialorbitofrontal | 183.266 | 33.521 | 5.467 | 0.000 | 117.111 | 249.422 | **0.000** | 0.817 |
| R_middletemporal | 422.789 | 63.971 | 6.609 | 0.000 | 296.540 | 549.037 | **0.000** | 0.988 |
| R_parahippocampal | 56.622 | 12.715 | 4.453 | 0.000 | 31.529 | 81.716 | **0.001** | 0.666 |
| R_paracentral | 137.569 | 28.640 | 4.803 | 0.000 | 81.047 | 194.090 | **0.000** | 0.718 |
| R_parsopercularis | 173.435 | 29.716 | 5.836 | 0.000 | 114.789 | 232.082 | **0.000** | 0.873 |
| R_parsorbitalis | 109.609 | 15.982 | 6.858 | 0.000 | 78.069 | 141.149 | **0.000** | 1.025 |
| R_parstriangularis | 234.733 | 35.727 | 6.570 | 0.000 | 164.226 | 305.241 | **0.000** | 0.982 |
| R_pericalcarine | 150.252 | 40.265 | 3.732 | 0.000 | 70.787 | 229.717 | **0.017** | 0.558 |
| R_postcentral | 411.450 | 69.269 | 5.940 | 0.000 | 274.745 | 548.155 | **0.000** | 0.888 |
| R_posteriorcingulate | 115.778 | 26.444 | 4.378 | 0.000 | 63.589 | 167.966 | **0.001** | 0.655 |
| R_precentral | 416.285 | 69.863 | 5.959 | 0.000 | 278.407 | 554.163 | **0.000** | 0.891 |
| R_precuneus | 479.638 | 74.809 | 6.411 | 0.000 | 332.000 | 627.277 | **0.000** | 0.959 |
| R_rostralanteriorcingulate | 72.482 | 17.204 | 4.213 | 0.000 | 38.529 | 106.435 | **0.003** | 0.630 |
| R_rostralmiddlefrontal | 840.199 | 121.796 | 6.898 | 0.000 | 599.832 | 1080.567 | **0.000** | 1.031 |
| R_superiorfrontal | 855.068 | 136.426 | 6.268 | 0.000 | 585.826 | 1124.310 | **0.000** | 0.937 |
| R_superiorparietal | 674.962 | 103.993 | 6.490 | 0.000 | 469.728 | 880.197 | **0.000** | 0.970 |
| R_superiortemporal | 417.658 | 65.306 | 6.395 | 0.000 | 288.775 | 546.541 | **0.000** | 0.956 |
| R_supramarginal | 444.089 | 84.320 | 5.267 | 0.000 | 277.680 | 610.498 | **0.000** | 0.787 |
| R_frontalpole | 35.695 | 7.053 | 5.061 | 0.000 | 21.776 | 49.614 | **0.000** | 0.757 |
| R_temporalpole | 33.537 | 10.407 | 3.223 | 0.002 | 12.998 | 54.076 | 0.103 | 0.482 |
| R_transversetemporal | 29.221 | 6.357 | 4.596 | 0.000 | 16.674 | 41.767 | **0.001** | 0.687 |
| R_insula | 217.943 | 38.531 | 5.656 | 0.000 | 141.901 | 293.985 | **0.000** | 0.846 |

Table S6. B) Cortical area difference between genders adjusted for age and ICV.

| **Variable** | **estimate** | **std.error** | **statistic** | **p.value** | **conf.low** | **conf.high** | **p_adj** | **Cohen_d** |
| --- | --- | --- | --- | --- | --- | --- | --- | --- |
| L_bankssts | 84.644 | 27.368 | 3.093 | 0.002 | 30.630 | 138.657 | 0.157 | 0.470 |
| L_caudalanteriorcingulate | 29.327 | 19.992 | 1.467 | 0.144 | -10.130 | 68.784 | 1.000 | 0.223 |
| L_caudalmiddlefrontal | 258.009 | 55.613 | 4.639 | 0.000 | 148.250 | 367.768 | **0.000** | 0.705 |
| L_cuneus | 143.912 | 35.410 | 4.064 | 0.000 | 74.026 | 213.798 | **0.005** | 0.617 |
| L_entorhinal | 42.327 | 15.740 | 2.689 | 0.008 | 11.263 | 73.391 | 0.534 | 0.408 |
| L_fusiform | 340.622 | 55.987 | 6.084 | 0.000 | 230.126 | 451.118 | **0.000** | 0.924 |
| L_inferiorparietal | 385.399 | 103.426 | 3.726 | 0.000 | 181.277 | 589.522 | **0.018** | 0.566 |
| L_inferiortemporal | 375.907 | 66.060 | 5.690 | 0.000 | 245.531 | 506.283 | **0.000** | 0.864 |
| L_isthmuscingulate | 81.072 | 23.731 | 3.416 | 0.001 | 34.236 | 127.909 | 0.054 | 0.519 |
| L_lateraloccipital | 498.960 | 87.880 | 5.678 | 0.000 | 325.518 | 672.401 | **0.000** | 0.862 |
| L_lateralorbitofrontal | 221.863 | 42.041 | 5.277 | 0.000 | 138.890 | 304.837 | **0.000** | 0.801 |
| L_lingual | 288.664 | 57.080 | 5.057 | 0.000 | 176.010 | 401.317 | **0.000** | 0.768 |
| L_medialorbitofrontal | 142.387 | 34.946 | 4.074 | 0.000 | 73.417 | 211.357 | **0.005** | 0.619 |
| L_middletemporal | 314.901 | 63.929 | 4.926 | 0.000 | 188.731 | 441.072 | **0.000** | 0.748 |
| L_parahippocampal | 43.399 | 12.734 | 3.408 | 0.001 | 18.267 | 68.531 | 0.055 | 0.518 |
| L_paracentral | 100.753 | 25.050 | 4.022 | 0.000 | 51.315 | 150.191 | **0.006** | 0.611 |
| L_parsopercularis | 174.256 | 40.914 | 4.259 | 0.000 | 93.507 | 255.005 | **0.002** | 0.647 |
| L_parsorbitalis | 91.895 | 14.106 | 6.514 | 0.000 | 64.055 | 119.736 | **0.000** | 0.989 |
| L_parstriangularis | 139.877 | 36.654 | 3.816 | 0.000 | 67.536 | 212.219 | **0.013** | 0.579 |
| L_pericalcarine | 123.006 | 37.928 | 3.243 | 0.001 | 48.152 | 197.861 | 0.096 | 0.492 |
| L_postcentral | 486.650 | 79.485 | 6.123 | 0.000 | 329.777 | 643.522 | **0.000** | 0.930 |
| L_posteriorcingulate | 121.463 | 25.998 | 4.672 | 0.000 | 70.153 | 172.773 | **0.000** | 0.709 |
| L_precentral | 512.772 | 73.221 | 7.003 | 0.000 | 368.263 | 657.282 | **0.000** | 1.063 |
| L_precuneus | 444.220 | 76.316 | 5.821 | 0.000 | 293.603 | 594.838 | **0.000** | 0.884 |
| L_rostralanteriorcingulate | 66.249 | 24.714 | 2.681 | 0.008 | 17.474 | 115.024 | 0.547 | 0.407 |
| L_rostralmiddlefrontal | 706.211 | 105.335 | 6.704 | 0.000 | 498.320 | 914.102 | **0.000** | 1.018 |
| L_superiorfrontal | 845.738 | 131.059 | 6.453 | 0.000 | 587.078 | 1104.398 | **0.000** | 0.980 |
| L_superiorparietal | 464.243 | 115.753 | 4.011 | 0.000 | 235.790 | 692.695 | **0.006** | 0.609 |
| L_superiortemporal | 431.477 | 66.878 | 6.452 | 0.000 | 299.486 | 563.467 | **0.000** | 0.980 |
| L_supramarginal | 540.018 | 110.700 | 4.878 | 0.000 | 321.538 | 758.497 | **0.000** | 0.741 |
| L_frontalpole | 25.828 | 5.423 | 4.762 | 0.000 | 15.125 | 36.532 | **0.000** | 0.723 |
| L_temporalpole | 26.197 | 10.015 | 2.616 | 0.010 | 6.431 | 45.964 | 0.658 | 0.397 |
| L_transversetemporal | 44.448 | 9.809 | 4.531 | 0.000 | 25.088 | 63.808 | **0.001** | 0.688 |
| L_insula | 137.970 | 38.372 | 3.596 | 0.000 | 62.239 | 213.701 | **0.029** | 0.546 |
| R_bankssts | 91.770 | 19.323 | 4.749 | 0.000 | 53.633 | 129.907 | **0.000** | 0.721 |
| R_caudalanteriorcingulate | 48.878 | 20.360 | 2.401 | 0.017 | 8.696 | 89.060 | 1.000 | 0.365 |
| R_caudalmiddlefrontal | 161.985 | 53.011 | 3.056 | 0.003 | 57.361 | 266.609 | 0.177 | 0.464 |
| R_cuneus | 148.154 | 33.063 | 4.481 | 0.000 | 82.901 | 213.407 | **0.001** | 0.680 |
| R_entorhinal | 56.259 | 13.351 | 4.214 | 0.000 | 29.910 | 82.608 | **0.003** | 0.640 |
| R_fusiform | 349.097 | 51.698 | 6.753 | 0.000 | 247.066 | 451.128 | **0.000** | 1.025 |
| R_inferiorparietal | 600.013 | 124.109 | 4.835 | 0.000 | 355.071 | 844.956 | **0.000** | 0.734 |
| R_inferiortemporal | 367.404 | 61.201 | 6.003 | 0.000 | 246.617 | 488.191 | **0.000** | 0.912 |
| R_isthmuscingulate | 83.640 | 22.552 | 3.709 | 0.000 | 39.132 | 128.149 | **0.019** | 0.563 |
| R_lateraloccipital | 536.513 | 83.180 | 6.450 | 0.000 | 372.349 | 700.678 | **0.000** | 0.979 |
| R_lateralorbitofrontal | 211.551 | 51.558 | 4.103 | 0.000 | 109.795 | 313.307 | **0.004** | 0.623 |
| R_lingual | 241.698 | 63.720 | 3.793 | 0.000 | 115.938 | 367.457 | **0.014** | 0.576 |
| R_medialorbitofrontal | 162.620 | 32.954 | 4.935 | 0.000 | 97.581 | 227.659 | **0.000** | 0.749 |
| R_middletemporal | 390.898 | 63.680 | 6.138 | 0.000 | 265.219 | 516.578 | **0.000** | 0.932 |
| R_parahippocampal | 51.858 | 12.786 | 4.056 | 0.000 | 26.624 | 77.092 | **0.005** | 0.616 |
| R_paracentral | 128.379 | 28.899 | 4.442 | 0.000 | 71.344 | 185.413 | **0.001** | 0.675 |
| R_parsopercularis | 158.691 | 29.588 | 5.363 | 0.000 | 100.296 | 217.086 | **0.000** | 0.814 |
| R_parsorbitalis | 98.072 | 15.494 | 6.330 | 0.000 | 67.493 | 128.652 | **0.000** | 0.961 |
| R_parstriangularis | 215.739 | 35.449 | 6.086 | 0.000 | 145.776 | 285.702 | **0.000** | 0.924 |
| R_pericalcarine | 131.913 | 40.237 | 3.278 | 0.001 | 52.500 | 211.326 | 0.086 | 0.498 |
| R_postcentral | 384.991 | 69.619 | 5.530 | 0.000 | 247.591 | 522.391 | **0.000** | 0.840 |
| R_posteriorcingulate | 103.572 | 26.412 | 3.921 | 0.000 | 51.445 | 155.698 | **0.009** | 0.595 |
| R_precentral | 380.505 | 69.455 | 5.478 | 0.000 | 243.427 | 517.583 | **0.000** | 0.832 |
| R_precuneus | 449.976 | 75.108 | 5.991 | 0.000 | 301.742 | 598.210 | **0.000** | 0.910 |
| R_rostralanteriorcingulate | 66.162 | 17.309 | 3.822 | 0.000 | 32.001 | 100.322 | **0.012** | 0.580 |
| R_rostralmiddlefrontal | 755.413 | 118.509 | 6.374 | 0.000 | 521.521 | 989.304 | **0.000** | 0.968 |
| R_superiorfrontal | 746.349 | 130.757 | 5.708 | 0.000 | 488.284 | 1004.414 | **0.000** | 0.867 |
| R_superiorparietal | 623.367 | 103.543 | 6.020 | 0.000 | 419.012 | 827.722 | **0.000** | 0.914 |
| R_superiortemporal | 364.950 | 62.488 | 5.840 | 0.000 | 241.624 | 488.276 | **0.000** | 0.887 |
| R_supramarginal | 407.799 | 84.437 | 4.830 | 0.000 | 241.154 | 574.445 | **0.000** | 0.733 |
| R_frontalpole | 32.316 | 7.033 | 4.595 | 0.000 | 18.434 | 46.197 | **0.001** | 0.698 |
| R_temporalpole | 30.640 | 10.526 | 2.911 | 0.004 | 9.867 | 51.414 | 0.277 | 0.442 |
| R_transversetemporal | 25.967 | 6.321 | 4.108 | 0.000 | 13.493 | 38.441 | **0.004** | 0.624 |
| R_insula | 196.672 | 38.151 | 5.155 | 0.000 | 121.376 | 271.968 | **0.000** | 0.783 |
